# Mesenchymal Stem Cell Extracellular Vesicles from Tissue-Mimetic System Enhance Epidermal Regeneration via Formation of Migratory Cell Sheets

**DOI:** 10.1007/s13770-023-00565-6

**Published:** 2023-07-29

**Authors:** Jacob G. Hodge, Jennifer L. Robinson, Adam J. Mellott

**Affiliations:** 1 Bioengineering Graduate Program, University of Kansas, Lawrence, KS USA; 2 Department of Plastic Surgery, University of Kansas Medical Center, 3901 Rainbow Blvd., Mail Stop: 3051, Kansas City, KS USA; 3 Department of Chemical and Petroleum Engineering, University of Kansas, Lawrence, KS USA; 4 Department of Orthopaedics and Sports Medicine, University of Washington, Seattle, WA USA; 5 Department of Mechanical Engineering, University of Washington, Seattle, WA USA; 6 Institute for Stem Cell and Regenerative Medicine, University of Washington, Seattle, WA USA; 7 Ronawk Inc., Olathe, KS USA

**Keywords:** Epidermal regeneration, Exosomes, Wound healing

## Abstract

**Background:**

The secretome of adipose-derived mesenchymal stem cells (ASCs) offers a unique approach to understanding and treating wounds, including the critical process of epidermal regeneration orchestrated by keratinocytes. However, 2D culture techniques drastically alter the secretory dynamics of ASCs, which has led to ambiguity in understanding which secreted compounds (e.g., growth factors, exosomes, reactive oxygen species) may be driving epithelialization.

**Methods:**

A novel tissue-mimetic 3D hydrogel system was utilized to enhance the retainment of a more regenerative ASC phenotype and highlight the functional secretome differences between 2D and 3D. Subsequently, the ASC-secretome was stratified by molecular weight and the presence/absence of extracellular vesicles (EVs). The ASC-secretome fractions were then evaluated to assess for the capacity to augment specific keratinocyte activities.

**Results:**

Culture of ASCs within the tissue-mimetic system enhanced protein secretion ~ 50%, exclusively coming from the > 100 kDa fraction. The ASC-secretome ability to modulate epithelialization functions, including migration, proliferation, differentiation, and morphology, resided within the “> 100 kDa” fraction, with the 3D ASC-secretome providing the greatest improvement. 3D ASC EV secretion was enhanced two-fold and exhibited dose-dependent effects on epidermal regeneration. Notably, ASC-EVs induced morphological changes in keratinocytes reminiscent of native regeneration, including formation of stratified cell sheets. However, only 3D-EVs promoted collective cell sheet migration and an epithelial-to-mesenchymal-like transition in keratinocytes, whereas 2D-EVs contained an anti-migratory stimulus.

**Conclusion:**

This study demonstrates how critical the culture environment is on influencing ASC-secretome regenerative capabilities. Additionally, the critical role of EVs in modulating epidermal regeneration is revealed and their translatability for future clinical therapies is discussed.

**Supplementary Information:**

The online version contains supplementary material available at 10.1007/s13770-023-00565-6.

## Introduction

Skin is the largest organ of the human body and provides a crucial external barrier for our body [1]. Repair and regeneration of the skin is a dynamic series of events that occurs in the setting of tissue damage and involves a diverse array of interrelated cell populations that communicate via mechanical, physical, and biochemical cues [2, 3]. Secreted paracrine compounds from resident cell populations maintain a key role in orchestrating proper cellular and molecular signaling pathways during the wound healing process. Ultimately, the goal of physiological wound healing is to promote the migration and proliferation of appropriate cells into the wound environment, stimulate neotissue formation via deposition and remodeling of extracellular matrix (ECM) components, and modulate cellular biophysical and biomechanical constructs (e.g., cytoskeletal rearrangements to promote migration and/or wound contraction) [3, 4].

During physiological wound healing, cells undergo phenotypic changes to increase their regenerative capabilities in an attempt to restore anatomical homeostasis. Notably, a fundamental step of proper wound healing is sufficient “closure” of the wound, also known as re-epithelialization, which helps protect the wound and deeper tissue structures [5]. The process of re-epithelialization is performed by epidermal keratinocytes, and inadequate keratinocyte activity will result in protraction of wound closure and an increased risk for adverse outcomes such as chronic wounds, surgical interventions, need for amputations, and an overall increase in patient morbidity [5, 6]. Proper keratinocyte re-epithelialization relies on keratinocytes modulating their cytoskeletal and junctional proteins to become more migratory and/or proliferative. Paracrine activity from surrounding cell populations in the epidermis, dermis, hair follicles and subcutaneous tissue play a key role in orchestrating this phenotypic switch of keratinocytes [5]. However, there remains a critical need to develop therapies aimed at augmenting appropriate keratinocyte functionality to prevent progression towards chronic wounds.

Recent investigations into stem cell derived therapies have emerged as possible avenues for producing future regenerative treatments for wounds [7–9]. More specifically, mesenchymal stem/stromal cells (MSCs) are multipotent progenitor cells that are found in a variety of tissue sources within the human body including dermal adipose and hair follicles and contain intrinsic regenerative capabilities due to the compositional plasticity of their secretory profile [10, 11]. Recent research has demonstrated that MSCs can exhibit diverse therapeutic capabilities for enhancing wound healing via modulation of a number of tissue regenerative processes [11]. Notably, recent studies suggest that the adaptive secretory nature of MSCs, rather than their multipotent nature, may be driving many of the regenerative effects seen in prior *in vitro* and *in vivo* studies [12–14]. Thus, MSC-derived acellular byproducts offer a cell-free alternative to current cell-based therapies and have shown the capacity to modulate wound healing activity, including the augmentation of keratinocyte re-epithelialization activity.

Interestingly, prior clinical studies investigating the utility of biological compounds, such as growth factors or cytokines, have demonstrated mixed results [15, 16]. However, this is likely due to the more complex nature of wounds (especially chronic wounds) that require a heterogenous milieu of factors that balance one another rather than the presence/absence of a single compound (e.g., single growth factor). Additionally, a growing body of evidence suggests extracellular vesicles (EVs), such as exosomes, are more stable and may play a key role in native tissue signaling and regeneration [17–19]. EVs consist of vesicle-like particles released from cells and include apoptotic bodies, microvesicles (MVs), and exosomes [20]. EV activity and quality is dependent on the dynamic array of biomodulatory compounds found within them, which can change depending on external stimuli and cellular phenotype. Notably, the secretome of adipose-derived MSCs (ASCs) has demonstrated the ability to enhance the rate of wound healing and tissue regeneration *in vivo* [21–23]. However, to date, it is still not clear what fractions of MSC-derived secretory factors are driving which regenerative wound healing activities, such as augmented keratinocyte migratory and proliferative activity, seen in prior studies. This is in part, likely due to the inconsistent and inefficient utilization of standard 2D culture modalities that result in MSC populations with variable phenotypes and regenerative capabilities, as well as the complex and diverse nature of the MSC secretome. Thus, investigations into more robust tissue-mimetic 3D systems could provide a platform to standardize MSC culture and generate more rigorous data pertaining to the regenerative mechanisms of MSC-derived biologics and provide an opportunity to enhance and/or tailor their regenerative capabilities.

It is important to understand that our body’s native tissue microenvironments provide a range of biomechanical and biophysical cues that regulate cellular fate and behavior within that environmental niche [24–26]. Native 3D tissue environments typically exhibit a range of viscoelastic properties that affect mechanotransductive and biochemical pathways [27, 28]. MSCs have been shown to be highly dependent upon these 3D interactions, including the role of focal adhesive sites, cell-to-cell interfaces, applied forces, and matrix stiffness, to maintain their “stem-like” phenotype and regenerative capabilities. Thus, the native MSC niche is more accurately represented by *in vitro* 3D systems rather than 2D, which can improve the efficacy and reproducibility of future regenerative therapies and provide a more appropriate understanding of physiological signaling [29–32]. Notably, most current 3D hydrogel systems rely on manipulating densely-packed crosslinked networks in order to modulate the mechanical properties of the hydrogels, which drastically limits the ability for molecules to readily diffuse throughout the system [24, 33, 34]. However, the novel microchannel design of the 3D hydrogel system in this study acts as a “pseudo-vascular” conduit and does not hinder mass transport like traditional poured/molded hydrogel systems, which improved the collection of secreted byproducts, in addition to adequate nutrient exchange and prevention of centralized necrosis within the hydrogel [31].

Barriers related to the translatability of cell-based regenerative therapies, such as depleted regenerative capabilities of unhealthy autologous populations and inadequate ex vivo expansion systems, have led to a rising interest in regenerative acellular therapies [35]. While previous data with MSC-derived acellular products are promising, experiments using MSCs in wound healing to date have mostly utilized standard 2D culture techniques, which are known to induce differentiation and senescence [31, 32, 36]. Thus, traditional 2D culture modalities likely result in a heterogeneous and less regenerative MSC populations, leading to impurities and/or an inconsistent secretive product that subsequently limits the potential clinical benefits of MSC therapies. As a result, there is a growing interest in developing more efficient 3D expansion systems.

In this study, we utilize a tissue-mimetic 3D hydrogel system that is mechanically analogous to native adipose tissue for the culture of ASCs (Table 1). We have previously published the benefits of this novel system in protecting ASC populations from induction of senescence and loss of a “stem-like” phenotype, subsequently resulting in a more regenerative population capable of modulating wound healing activity [31, 37]. An additional benefit of this system is the unique microarchitectural design that does not hinder mass transport and permits the efficient collection of secreted byproducts. Thus, this system acts like a “bioreactor” for generating biological byproducts from cell populations. Within this study, we stratify ASC conditioned media (ASC-CM) based on molecular weight cutoffs and EV/exosome particle content in order to compare and identify which fractions of ASC-CM are driving specific wound healing activities previously seen in keratinocytes. Uniquely, we will compare and contrast the effect of ASC-CM from 2D versus 3D. We hypothesized that the culture conditions of our tissue-mimetic system would be a more effective measure of how ASCs respond natively and identify functional differences between the ASC-CM from 2D and 3D. Moreover, we suspect that the exosome fraction of “stem-like” populations, such as ASCs, are critical in driving many of the functionals benefits seen in keratinocyte wound healing activity from prior studies.

**Table 1:** Mechanical and viscoelastic properties of the tissue-mimetic hydrogel and human adipose

Frequency obtained	Storage modulus (E′) kPa	Loss modulus (E′′) kPa	TanDelta (E′′/E′)	"Secant" modulus (E) (at plastic deformation)
	1 Hz			
Bio-block hydrogel system	48.5	4.5	0.1	8.4
Native adipose (based on literature)	10–100	1–20	0.1–0.2	1–10

## Materials and methods

### Cell culture

Human adipose-derived mesenchymal stem cells (ASCs; Lonza, Basel, Swiss; Lot #18TL212639, 23-year-old Female, Black), human keratinocytes (KCs; Lonza, Lot #18TL318559, 62-year-old Male, Caucasian), were utilized in this study. Younger ASCs and older KCs were desired for this study to better replicate a potential clinical scenario of a healthy-sourced allogeneic ASC-derived wound therapy, due to the tendency for younger KCs to perform regenerative healing more efficiently than older KCs. ASCs were cultured in RoosterNourish MSC-XF (RoosterBio, Frederick, MD, USA; Cat. #KT-016) for growth media (MSC-GM) and switched to RoosterCollect EV-Pro (RoosterBio; Cat. #K41001) for serum-free, low particulate media. DermaLife K Keratinocyte Medium Complete Kit was obtained from Lifeline Cell Technologies (Maryland, USA; #LL-0007) and used for KC culture.

### Three-dimensional (3D) printed hydrogel cell culture system

The 3D hydrogel system is ~ 1-cm^3^ and is a bioprinted cell culture and expansion system called an X-Block (Ronawk Inc., Kansas, USA) that contains a unique macro- and micro-architectural design that permits mass transport and nutrient exchange. The hydrogel is printed utilizing a pre-defined and specific microstructure that results in the creation of voided/porous regions that create continuous microchannels with 300-µm diameters. The microporous component makes up ~ 44% of the total volume of the hydrogel. Additionally, the integrated microarchitectural design significantly increases the surface area-to-volume ratio to enhance cellular proliferation and migration. The X-Block hydrogels, (Ronawk Inc.) are fabricated with a proprietary mixture of biodegradable substrates that contain biologically native binding epitopes for cellular attachment. The 3D hydrogels were placed into a glass 6-well culture plate for culturing. Cells were then added dropwise to the surface of the hydrogels and allowed to migrate into the microarchitecture over the span of 15-min, followed by submersion of the hydrogels with culture media. A thin coating of the bioink was utilized for the 2D culture control to account for any potential role of the substrate effects in 3D.

### Mechanical properties of tissue-mimetic hydrogel

A subset of 3D hydrogels were bioprinted at a z-height of 1.2-mm (thickness) while still maintaining all other dimensional and structural characteristics of the unique architectural design of the full-sized hydrogels. Hydrogels were analyzed with a Dynamic Mechanical Analyzer (RSA3; TA Instruments) setup to assess mechanical and viscoelastic properties (Table 1). A 5-mm biopsy punch was used to isolate a circular hydrogel sample to prevent force-concentrating points. Two (2) 5-mm punches were taken from each hydrogel and a total of four (4) hydrogel were evaluated for a total of eight (8) runs (n = 4). DMA was performed via a dynamic cylindrical compression analysis with a rate of compression of 0.005-mm/sec, in addition to a frequency sweep analysis over a range of 0.1–10 Hz. The frequency sweep analysis helps evaluate the viscoelastic properties of a given material by determining the relationship between a given compression frequency range and the storage (E′) and loss (E′′) moduli of a material. The compression frequency will gradually increase and the viscoelastic response at each given frequency is recorded. Expression of the viscoelastic moduli at 1 Hz is generally depicted due to the similarities of this frequency range and most soft tissues within physiological conditions, including human adipose [28, 38].

### Expansion of ASCs and KCs

“Passage 1 (P1)” ASCs and KCs were seeded on 2D plastic and cultured until ~ 80% confluency before subculturing. Subculturing of cells was performed by removing culture media, washing 3x, and incubating with 0.05% Trypsin/EDTA (Lonza; Cat. #CC-3232) at 37 °C for 5-min. Trypsin was neutralized with serum-based media and cells were centrifuged at 500 g for 5-min, pelleted, and resuspended for reseeding on new 2D tissue culture plastic vessels or for use in experimental assays. The increased surface area of a single 3D hydrogel eliminated the need for subculturing for the time course of this study (see prior study details), therefore 2D and 3D cells were seeded at the same seeding density of ~ 1,500 cells/cm^2^ to allow for analogous comparison between 2D and 3D culture. KCs were seeded at density of ~ 7500 cells/cm^2^ for experimental assays. After an initial characterization of P1 ASCs, subcultured ASCs at P2 were seeded into 3D or re-plated in 2D and cultured for 1-week and then analyzed or subcultured. 2D ASCs that were subcultured at 1-week were then re-plated for an additional week to obtain data for the P3 (2-week) data, whereas 3D ASCs were allowed to continuously culture within the 3D system without subculture (P3-equivalent/2-week) (n = 4).

### Assessment of ASC phenotype

Initial assessment of adipogenic, chondrogenic, and osteogenic trilineage differentiation potential of ASCs (at P1) was performed via culture with differentiating media, according to the manufacturer’s instructions and as previously described. Adipogenic differentiation was performed using hMSC Adipogenic Differentiation BulletKit™ (Lonza; Cat. #PT-3004). Chondrogenic differentiation was performed using hMSC Chondrogenic Differentiation Medium BulletKit™ (Lonza; Cat. #PT-3003), and was supplemented with TGF-β3 (Lonza; PT-4124) at a concentration of 10 ng/mL. Osteogenic differentiation was performed using hMSC Osteogenic Differentiation Medium BulletKit™ (Lonza; Cat. #PT-3002). All primary antibodies were obtained from Abcam (Cambridge, UK) unless otherwise stated. Evaluation of ASC “stem-like” phenotype was performed with the initial population at P1 via positive immunolabeling for CD73/90/105, as previously described. In brief, ASCs at P1 were seeded in 2D, fixed with 4% paraformaldehyde, washed 3x, blocked with 1% donkey-serum, and immunolabeled for CD34 (ab81289), CD45 (ab40763), CD90 (ab181469), CD105 (ab231774). CD73 (Cat. #41–0200) was obtained from Invitrogen (Waltham, MA). Cells were counterstained with an immunofluorescent nuclear marker, Hoechst 33342 (Invitrogen; Cat. #H3570) and for some samples Alexa Fluor 488 Phalloidin (ThermoFisher; Cat. #A12379). Additionally, ASCs from P1 (baseline control), P2 (1-week), and P3 (2-week) for 2D culture and P2 ASCs from 3D that were cultured for 1-week (P2-equivalent) or 2-weeks (P3-equivalent) were assessed via an RT^2^ Profiler™ PCR Array for Human Mesenchymal Stem Cells (Qiagen, Valencia, CA, USA; Cat. #330231; PAHS-082ZC-24) to evaluate expression of 84 MSC and MSC-associated genes. RNA was isolated and purified via an RNeasy Mini Kit (Qiagen). Only RNA with a 260/280 ratio of > 1.8 were used for this study. Cycle threshold (Ct) values were recorded and analyzed via the Delta-Delta-Ct method (Table S1). Glyceraldehyde 3-phosphate dehydrogenase (GAPDH), Beta-actin (ACTB), and Beta-2-Microglobulin (B2M) were the endogenous control genes utilized by the array (n = 3).

### *Functional characterization of cells *via* spectroscopy*

Both ASCs and KCs were evaluated with spectroscopy. Assays were carried out per manufacturer’s instructions and as previously described. In short, plated cells were analyzed via PicoGreen fluorescence obtained at 435/535 nm (n = 4) to quantify DNA as a surrogate measurement of proliferation. PrestoBlue fluorescence was obtained at 560/590 nm (n = 4) and displayed as an average relative fluorescent unit (R.F.U.) of PrestoBlue per Hoechst signal (350/460 nm) to obtain approximate metabolic activity per cell. Similarly, MitoTracker™ Red CMXRos (Invitrogen; Cat #M7512; 500 nM) fluorescence at 570/605 nm (n = 4) was used to assess mitochondrial activity/membrane potential. Lastly, population doubling rate (PDR) of ASCs extracted from 2D or 3D, then re-plated into 2D (n = 5), and assessed via PicoGreen to obtain cell numbers at Day 1 and Day 4. The formula [(t)/((log(n/n_o_)) × 3.32], where t = time, n = cell number at final timepoint, n_o_ = initial cell number was used to calculate PDT.

### Assessment of ASC in situ proliferation in 2D and 3D

To evaluate the in situ proliferation of ASCs within the hydrogel system, 2D and 3D systems were seeded in parallel with the same seeding density and cell numbers were evaluated at initial seeding, 1-week, and 2-weeks. For 2D samples at 2-weeks, ASCs underwent one additional passaging event. At each respective time point ASCs were detached (2D) or isolated via hydrogel dissolution (3D) and assessed for cell number (PicoGreen). The X-Block hydrogel was dissolved with the Cell X-tract Solution (Cat. #R1R0100101-1), provided by Ronawk, that specifically acts on the substrate and does not target cellular proteins (n = 4).

### Isolation of ASC conditioned media

When conditioned medium (CM) from ASC culture was desired, MSC-GM media was removed, cells were washed 3× and serum-free MSC media was added for an additional 24-h wash. The 24-h wash was removed and new serum-free media was added followed by collections at 24-hr intervals for three consecutive days during days 6–8. Due to variability in 2D versus 3D proliferation rates, collection days were based on previously determined cell proliferation data to establish ratio of media/cell in order to standardize media consumption. 2D confluency was in the range of 60–80% and ASC-CM was only collected from the 1-week timepoint prior to any additional subculturing events in 2D. Collected ASC-CM was centrifuged at 1500 g for 10-min to eliminate cell debris, Steriflip filtered with a 0.22-μm filter, and stored at −80 °C for long-term storage until use. ASC-CM from each day (6, 7 or 8) for each replicate (n = 4) was combined to create a “batch” mixture to achieve adequate volumes for all experiments and eliminate any potential variability between ASC-CM from different days.

### ASC-CM stratification into molecular weight fractions

Collected ASC-CM underwent a series of centrifugation filtrations via 100 kDa, 30 kDa, 10 kDa, and 3 kDa molecular weight cutoff filters, starting with 100 kDa, per manufacturer’s instructions. Each centrifugation step was carried out at 4000 g for 30–45 min, the concentrate in the upper chamber was saved, and the filtrate in the lower chamber was collected and used for the next molecular weight filtration/centrifugation step until all that remained was a < 3 kDa filtrate. This resulted in an ASC-CM concentrate (~ 50–100x) for each molecular weight range.

### ASC-CM protein quantification

ASC-CM samples were quantified via total protein analysis with QuickDrop absorbance at 280 nm, BCA, and Bradford (Coomassie). Samples were then used for downstream analysis via protein quantification with a Pierce™ BCA Protein Assay Kit (Invitrogen; Cat. #23225), Pierce™ Coomassie “Bradford” Protein Assay Kit (Invitrogen; Cat. # 23200), and a QuickDrop (Molecular Devices; SpectraMax QuickDrop Micro-Volume Spectrophotometer) quantification via absorbance at 280-nm. Relative protein content was determined and back-calculated to determine what the relative concentration was within media before molecular weight concentration steps (e.g., 10-mL of media concentrated to 500-μL was a 20× concentration). Relative protein content for each molecular weight fraction was determined relative to “Full” ASC-CM before stratification. Assays were performed with technical and biological replicates (n = 4).

### ASC-CM extracellular vesicle (EV) production

EVs were isolated via ASC-CM centrifugation at 4000 g for 30-min through a 100 kDa centrifuge filter (same as above) followed by washing with PBS and re-centrifugation at 4000 g for 5-min through the 100 kDa filter, for a total of 3x washes. EVs were precipitated from the remaining “ > 100 kDa” concentrate overnight using a ExoQuick-TC kit (SBI; Cat. # EXOTC10A-1), per the manufacturer’s protocol. EV were resuspended in PBS and aliquots were removed and used to quantify protein content as an indirect measure of EV content via QuickDrop, BCA, and Bradford (Coomassie). Relative EV protein fraction was compared to total protein within “ > 100 kDa” and “Full” ASC-CM fractions. Additionally, purified EV samples were then evaluated via Nanoparticle Tracking Analysis (NTA; Malvern Panalytical; Nanosight LM10) to further determine both concentration and size distribution of particles extracted from the ASC-CM, with exosomes typically ranging from 25 to 250 nm (n = 4). Unused (not exposed to cells) serum-free ASC media underwent the same processing and was used to establish/determine baseline EV/particulate levels, which were negligible. EV particle counts of serum-free control ASC media were negligible and did not warrant a full workup, thus only a comparative analysis between 2 and 3D was fully conducted.

### KC functional activity after ASC-CM treatment

ASC-CM was used as a “supplement” for the Keratinocyte Growth Media (KC-GM) and dosed at a 2:1 ratio based on initial volume of ASC-CM (i.e., 20-mL of ASC-CM was concentrated to 200-μl and added to 10-mL of KC-GM). KCs were plated in 2D and allowed to acclimate and achieved desired confluences (> 24 hr). KC-GM was then removed, cells were washed, ASC-CM was applied for 24-hr, and experimental assays for metabolic, mitochondrial, proliferative, or migratory activity were then performed per the manufacturer’s instructions. Metabolic activity was assessed via PrestoBlue (as previously described, n = 4), proliferation was assessed via PicoGreen (as previously described, n = 4), mitochondrial activity was assessed via MitoTracker (as previously described, n = 4), and Migratory activity was assessed via a scratch “wound” assay. KC scratch assays were performed to evaluate changes in wound size/area as a surrogate measurement of KC migration after inflicted a scratch within a confluent monolayer of KCs (n = 4). Migration images were taken using an ImageXpress Micro XLS Imaging System (Molecular Devices) and the percent of wound area closed at 24-hr was determined via ImageJ analysis).

### KC senescence

KC senescence was performed via immunofluorescent labeling of β-galactosidase activity with the CellEvent™ Senescence Green Detection Kit (Invitrogen; Cat. #C10850), per manufacturer’s instructions. KCs from P2 or P3 were utilized for this study. KC-GM was removed from KC culture and ASC-CM stratified concentrates were dosed as a supplement to KC-GM for 24-h. KCs within KC-GM were used as a baseline measurement of senescence. After 24-hr, the KCs were fixed and stained. Hoechst 33342 was used as a counterstain to identify nuclei. Senescence characterization was carried out in quadruplicate (n = 4), with five (5) field of view images taken per biological replicate for a total of twenty (20) measurements per sample.

### KC Protein expression

Immunolabeling of cells was utilized for both ASCs and KCs. In brief, cells were washed 3× with HBSS, fixed with 4% PFA, and washed again 3× with HBSS. Cells were incubated in blocking buffer, which consisted of 2% donkey-serum with/without 0.1% Triton-X in HBSS, for at least 1-hr. Cells were then incubated with primary antibodies at 4$^\circ$C overnight. All primary antibodies were obtained from Abcam unless otherwise stated and included vimentin (ab8978), keratin 16 (ab76416), keratin 10 (ab76318), keratin 5 (ab52635). The next day cells were washed 3× with blocking buffer followed by application of secondary antibodies for 1-hr, then 3× washes with HBSS again. Cells were counterstained with an immunofluorescent nuclear marker, Hoechst 33342 and for some samples Alexa Fluor 488 Phalloidin. Secondary antibodies were derived in donkey and obtained from Invitrogen (n = 3).

### KC gene expression

RNA was isolated and purified as described in Sect. 2.4, in brief an RNeasy Mini Kit (Qiagen) was used according to manufacturer’s instructions. Untreated KCs cultured in KC-GM served as a control for KC analyses. Purity of cDNA samples was assessed with a QuickDrop spectrophotometer (Molecular Devices), with a 260/280 absorbance ratio > 1.8 was designated as pure. Individual qPCR primers (Qiagen; Cat. #330,001) were purchased to perform RT-qPCR on *CDKN2A, CDH1, CDH2, FLG, K10, K16, K5, TWIST1, VIM,* and *CCND1*. *GAPDH* was used as an endogenous control for these samples as well (n = 3).

### EV *in vitro* tracking

EVs previously isolated from the ASC-CM were fluorescently labeled with the lipophilic membrane stain, Dil (Invitrogen; Cat. #D282), at a concentration of 1-μM. The labeled EVs were washed with PBS and re-centrifuged at 3500 g for 15-min through the 100 kDa filter, for a total of 3x washes and centrifugations to remove excess dye. DiI-labeled EVs were added to KC-GM, which was then applied to KCs for 24-hr. Media was removed and KCs were washed 2x, fixed, counterstained with Hoechst 33342, and Alexa Fluor 488 Phalloidin and observed under a fluorescence microscope.

### Dosing of EVs for 2D versus 3D quality comparison

EVs isolated from 2D and 3D were then resuspended at known concentrations and added to KC-GM for a final concentration of 5, 25, or 250 μg/mL of EV protein within the KC-GM. EV concentration calculated based on average of all three protein analyses (QuickDrop, BCA, Bradford) (n = 4).

### Imaging analysis

Processing and analysis of images was performed with ImageJ and CellProfiler. All sets of images and image analyses were automated with a standardized pipeline and treated the same way across all similar image sets.

### Statistical analysis

All data were reported as means with standard error of mean (s.e.m.). Characterization analyses of ASC populations for metabolic, functional, and phenotypic data were evaluated with a One-way ANOVA. All Secretome stratification data were analyzed with a Two-way ANOVA. Characterization of extracellular vesicles (exosomes) was assessed via an unpaired student’s t-test. Exosome dosing studies were also evaluated with a Two-way ANOVA. A minimum of four replicates (n = 4) was used unless otherwise stated. Data were tested for normality via Shapiro–Wilk and Kolmogorov–Smirnov tests and plotted with a QQ plot. GraphPad Prism 9.4.2 software (La Jolla, CA) was used for the analyses and a *p* < 0.05 was considered significant. CellProfiler™ and ImageJ were utilized for image processing.

## Results

### Characterization of ASC populations

**Fig. 1:**
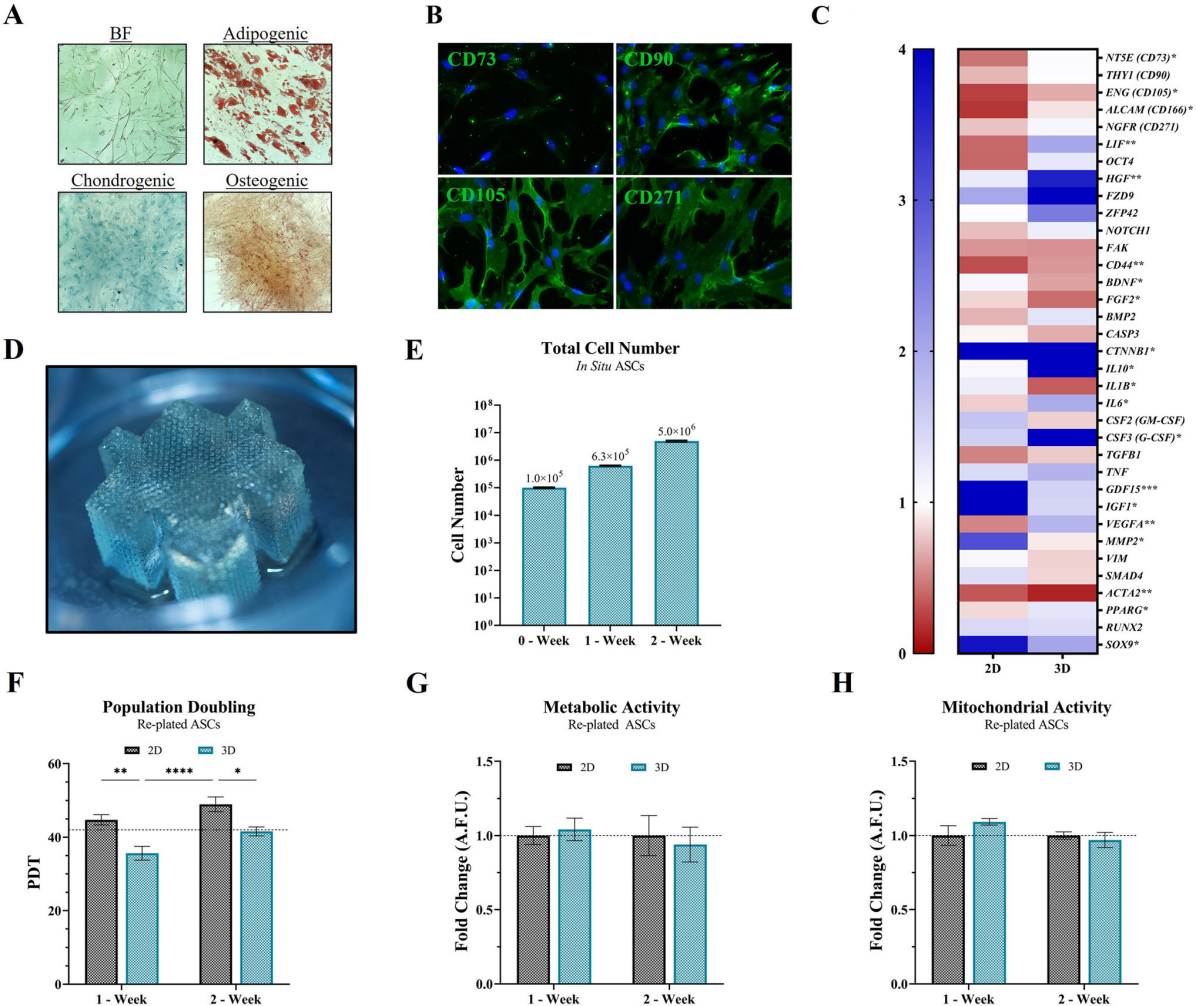
Characterization of ASC populations. Characterization of initial ASC P1 population with **A** Spindle/Mesenchymal-like adherent cells with trilineage/multipotent potential, **B** CD surface marker expression of CD73/90/105/271 [Scale bar = 100 μm; negative markers of CD34 and CD45 exhibited less than 5% positive − *data not shown*], and **C** Gene expression of key markers via MSC Phenotyping array. **D** Photograph of the 3D-printed, ~ 1cm^3^ tissue-mimetic X-Block within 6-well culture vessel. The X-Blocks are printed with a predefined microarchitecture that results in a substantial increase in surface area-to-volume via the formation of microchannels that result in ~ 44% porous/voided space within the macrostructure. Additionally, the X-Blocks were fabricated to mimic the mechanical and viscoelastic properties native adipose tissue. **E** In situ cell number quantification within 3D system (*X-Block*). ASCs were then extracted from 2D or 3D and re-plated in 2D for analysis after 1-week (P2 for 2D) and 2-week (P3 for 2D) to assess for **F** Population Doubling Time, **G** Metabolic activity, and **H** Mitochondrial activity (*membrane potential*). Black dashed line indicates initial P1 population. Error bars are s.e.m. Significance denoted as *p < 0.05, **p < 0.01, ***p < 0.001, and ****p < 0.0001, n = 4

According to the International Society for Cellular Therapy, the minimum criteria for a cell population to be considered MSCs are that they are adherent (Fig. 1A), exhibit trilineage multipotent potential (Fig. 1A), and express MSC “stem-like” surface markers (Fig. 1B), including positive staining for at least CD73/90/105. Similarly, ASCs were then cultured in 2D or 3D for one (1) week and assessed for MSC phenotypic gene expression relative to the initial ASC population (Fig. 1C). ASCs within the 3D culture (Fig. 1D) exhibited a higher retainment of key MSC and MSC-like markers, whereas cells in 2D demonstrated a significant decline (Fig. 1C). Additionally, an in situ population doubling experiment was performed to assess relative cells numbers in 2D and 3D to determine optimal ASC-CM collection for future experiments (Fig. 1E). ASC-CM was collected when 2D ASCs exhibited 60–80% confluency, based on prior literature, and determined to be on days 6–8 (Fig. S1).

ASCs in our tissue-mimetic 3D hydrogel system were isolated and re-plated into 2D and further assessed for functional alteration in proliferation, metabolic, or mitochondrial activity, relative to traditional 2D culture. ASCs in 3D retained a lower population doubling time (PDT) upon re-plating in 2D, indicating cells were able to proliferate more rapidly (Fig. 1F). Moreover, the metabolic (Fig. 1G) and mitochondrial (Fig. 1H) health and activity of re-plated ASCs demonstrated no significant differences in 2D or 3D, over the time course of two weeks in either system.

### Larger MW secretome fraction is key driver of KC wound healing activity

**Fig. 2:**
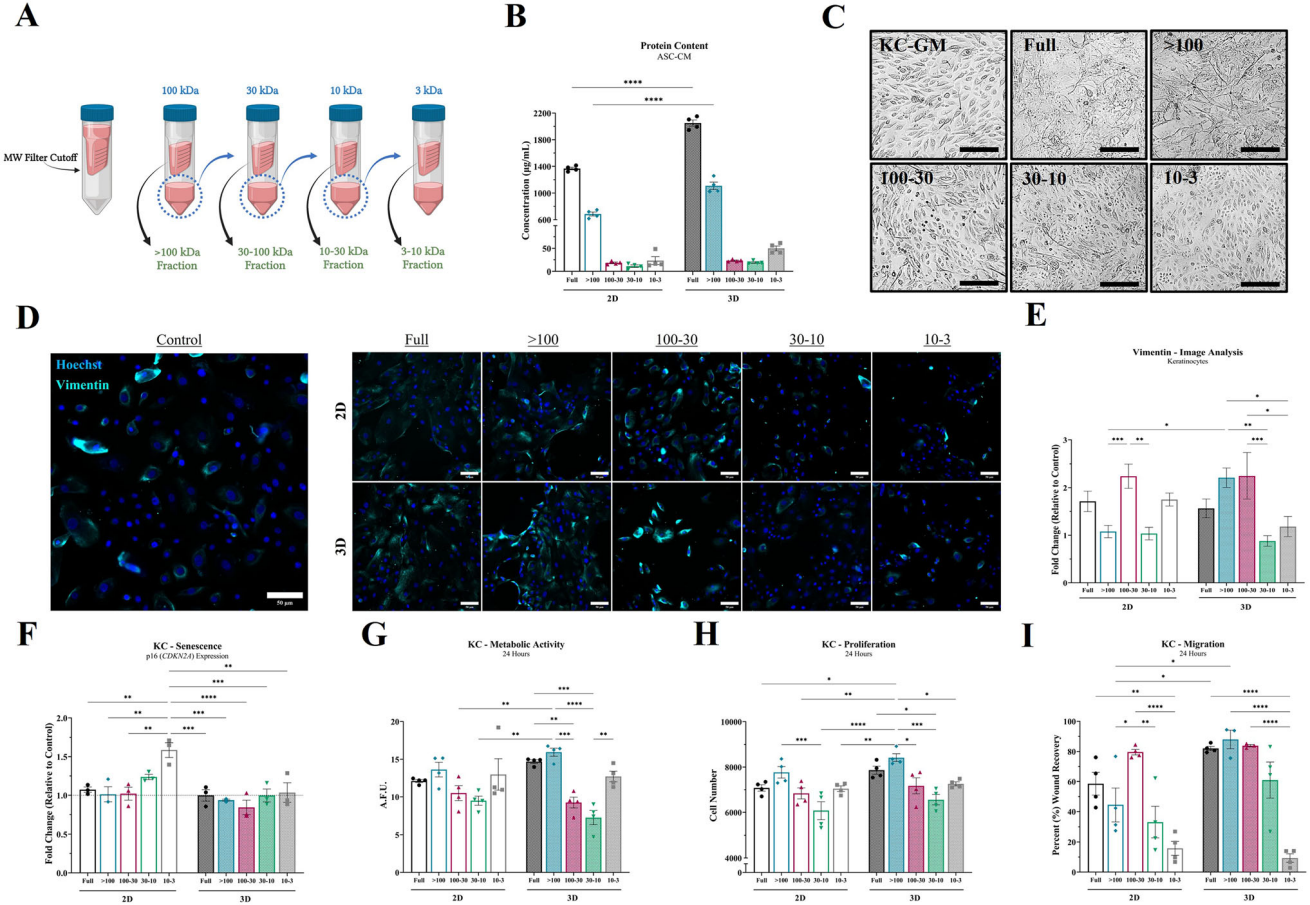
Larger MW secretome fraction is key driver of KC wound healing activity. **A** Schematic diagram of ASC-CM centrifugation filtration steps for each molecular weight (MW) kDa cutoff. Upper chamber solution considered concentrate for that range; residual lower chamber filtrate used for next MW filtration step. **B** ASC-CM protein content for each MW fraction. **C** Representative morphology images of KCs after treatment with KC-GM supplemented with different ASC-CM concentrates [Scale bar = 100 µm]. **D** Representative image panel of KCs treated with ASC-CM fractions from 2D or 3D and stained with Hoechst (Blue) and Vimentin (Cyan) [Scale bar = 50 µm]. **E** Image quantification of Vimentin immunolabeling of KCs (relative to baseline control KCs). **F** qRT-PCR analysis of p16^ink4a^ (senescence marker) of KCs after treatment with fractionated ASC-CM for 24 h. Dashed line is baseline control KCs. **G** Relative change in metabolic activity (via PrestoBlue). **H** Relative change in proliferation (via PicoGreen). **I** Relative change in migration (via scratch assay). Significance denoted as *p < 0.05, **p < 0.01, ***p < 0.001, and ****p < 0.0001, n = 4

ASC-CM collected from 2D and 3D was stratified by molecular weight (Fig. 2A) and each fraction was analyzed for total protein content (Fig. 2B) and ability to modulate a KC functional activity. ASCs within the tissue-mimetic system secreted ~ 40–50% more total protein than their 2D counterparts, where the “Full” and “100 kDa” fractions were the only groups significantly increased in 3D relative to 2D (Fig. 2B). All other tested fractions maintained a similar relative protein content between 2D and 3D (Fig. S2).

Previous studies have demonstrated that the ASC secretome can modulate the morphology of KCs to become more spindle-shaped and stratified, indicating a potential change in KC phenotype that may be important and related to the wound healing capacity of ASC-CM [37]. Control KCs (cultured with KC-GM) maintained a more homogenous rounded morphology, whereas the “100 kDa” fraction of ASC-CM promoted a morphological switch in KCs from both 2D and 3D culture towards a stratified cell sheet with spindle-like cells (Figs. 2C and S3). KCs treated with lower MW fractions of ASC-CM appeared to exhibit smaller cell sizes and a rounded morphology, without stratified cell sheets. Interestingly, KCs treated with higher MW fractions of ASC-CM, “100 kDa” and “100–30 kDa” fractions, demonstrated a significant increase in expression of the mesenchymal and migratory marker Vimentin (Fig. 2D, E). Conversely, KCs treated with lower MW fractions, from either 2D or 3D, exhibited increased expression of the senescence-related markers p16 and β-Galactosidase (Figs. 2F and S4).

The metabolic, proliferative, and migratory activity of KCs were utilized as surrogate measures of *in vitro* wound healing activity. The “Full” ASC-CM from this 3D system has demonstrated benefits, relative to 2D, in prior studies [31, 37]. The “100 kDa” ASC-CM fraction for both 2D and 3D demonstrated a similar trend relative to “Full”, in its ability to enhance KC metabolic (Fig. 2G) and proliferative (Fig. 2H) activity. Whereas only the 3D group of “100 kDa” retained the enhanced migratory activity of KCs (Fig. 2I), with the 2D “100 kDa” group exhibiting a slight decrease, relative to “Full”. Additionally, the “100–30 kDa” fraction appeared to drive KC migratory activity for both 2D and 3D (Fig. 2I). The relative activity of the lower MW fractions of ASC-CM have similar capabilities toward augmenting metabolic and proliferative activity in KCs, when comparing the 2D or 3D groups to their counterparts. However, lower MW fractions demonstrate less capacity to augment KC functional activity than the larger MW fractions overall (i.e., “100 kDa and “Full”).

### ASC populations within tissue-mimetic system favor secretion of EVs

**Fig. 3:**
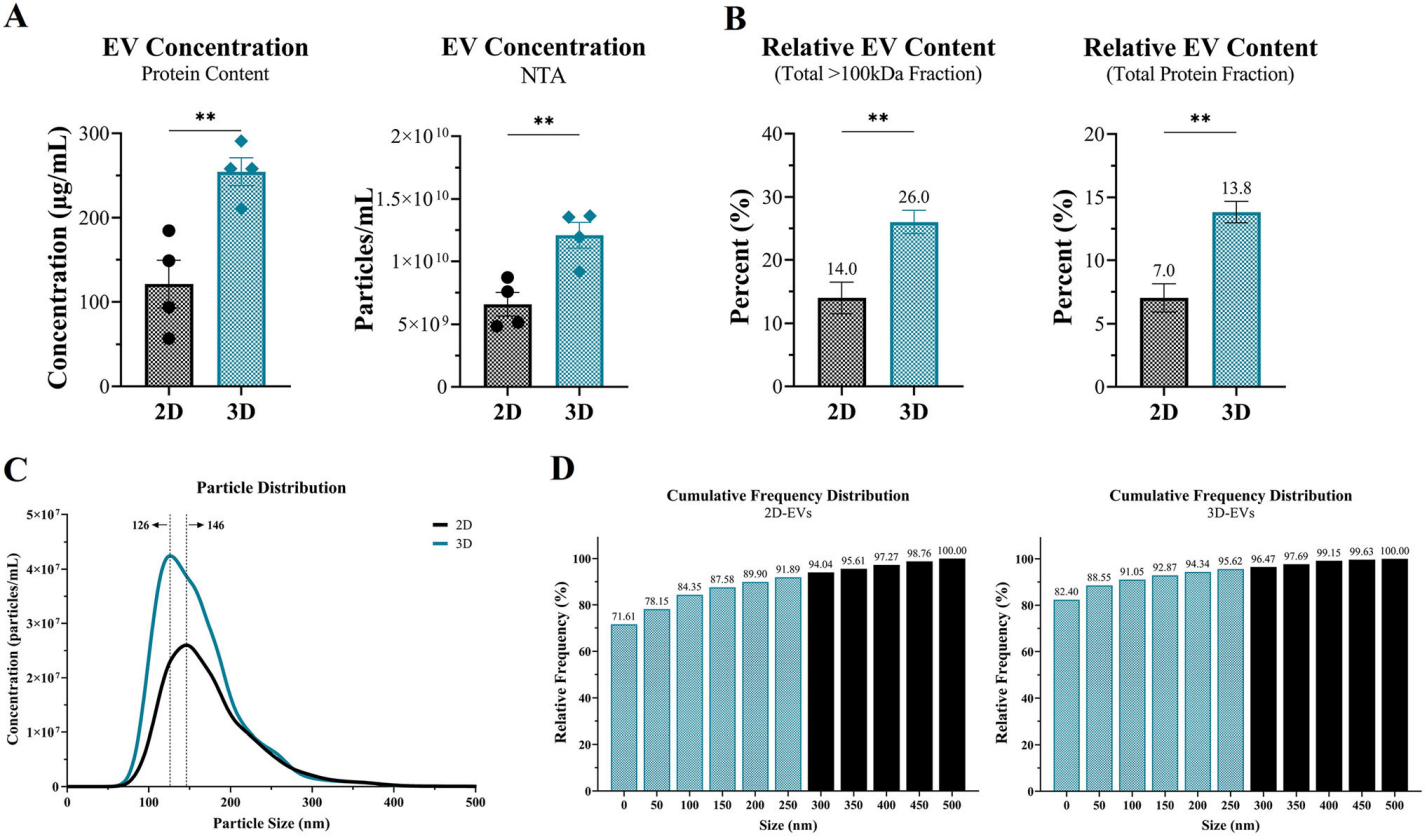
ASC populations within tissue-mimetic system favor secretion of EVs. **A** ASC-CM was processed for EV collection/isolation/purification from 2D and 3D culture and evaluated via protein content (QuickDrop/BCA/Bradford) of the EV fraction (*left*) and particle counts with NTA (*right*) to quantify EV concentrations. **B** Relative composition of EVs within ASC-CM was then quantified relative to protein content of “100 kDa” fraction (*left*) and total (i.e., “Full”) secreted protein content (*right*). **C** Analysis of EV size distribution for 2D and 3D was assessed with NTA data, and **D** Cumulative frequency distribution was generated to determine what percentage of measured particles fell within the standard exosome range (25–250 nm). Teal patterned bars indicate “within exosome size” range, whereas black bars are too large to likely be exosomes. Significance denoted as *p < 0.05, **p < 0.01, ***p < 0.001, and ****p < 0.0001, n = 4

Production and characterization of EV quantity and size distribution within ASC-CM was evaluated between 2D and 3D culture systems. Total EV production was increased over two-fold in 3D relative to 2D (Fig. 3A). Similarly, the relative secretion/composition of EV-to-Protein within the secretome was approximately 2:1, with ~ 14% (3D) or 7% (2D) of secreted protein being due to EVs (Fig. 3B). EVs were then characterized via nanoparticle tracking analysis (NTA) to determine size distribution of the EVs (Fig. 3C). NTA further validated an ~ two-fold increase in EV particles within 3D ASC-CM (Fig. 3A), in addition to > 90% of the particles exhibiting a size distribution within the range of exosomes (25–250 nm) (Fig. 3C, D). Notably, particles from 3D ASC-CM exhibited a trend towards a slightly smaller average size than 2D, but did not reach significance.

### ASC-EVs within tissue-mimetic system contain more potent re-epithelialization stimulus

**Fig. 4:**
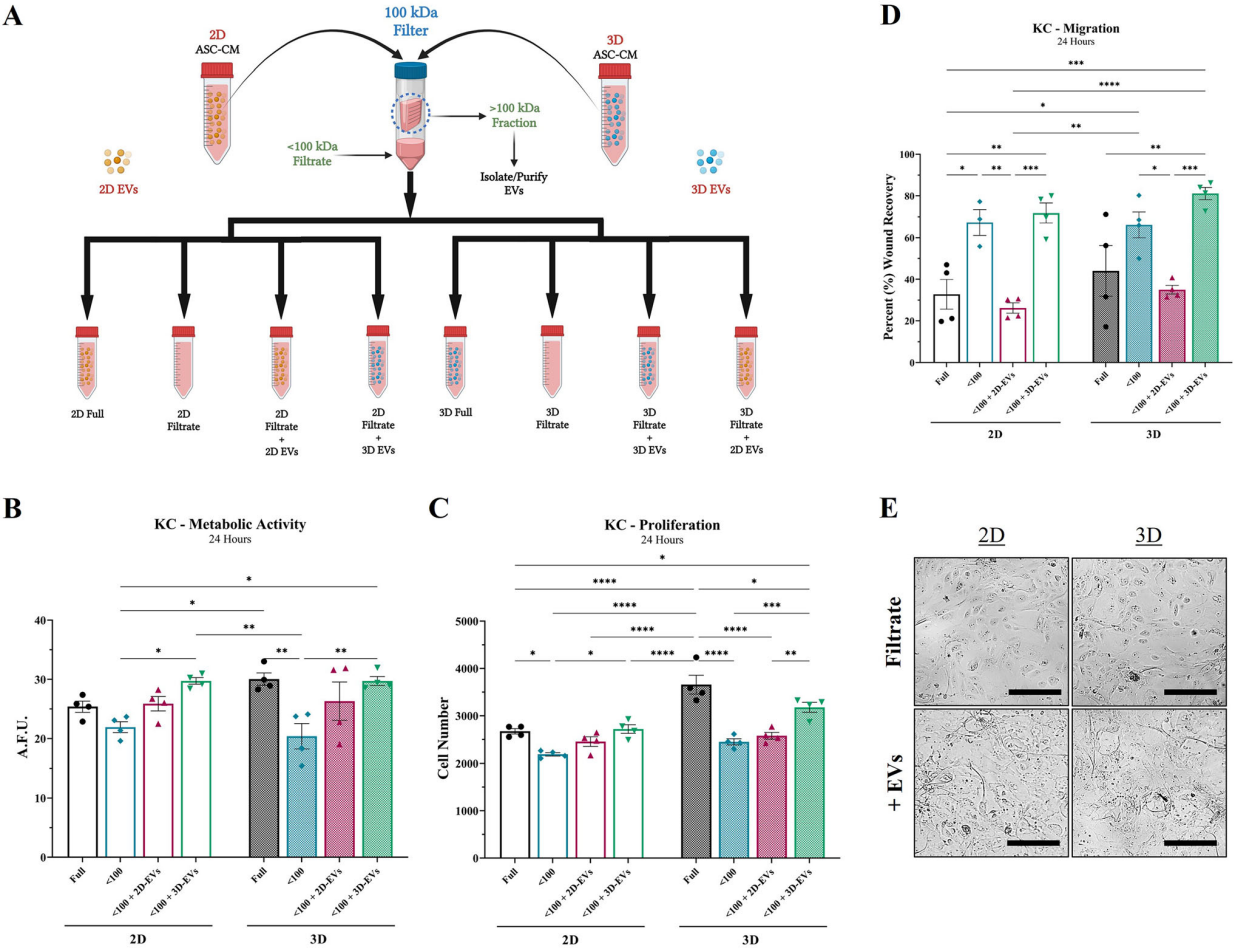
ASC-EVs within tissue-mimetic system contain more potent re-epithelialization stimulus. **A** Schematic diagram of ASC-CM filtration via 100-kDa centrifuge filter followed by subsequent EV/exosome isolation from the “100 kDa” concentrate in upper chamber. Isolated 2D-EVs (yellow circles) and 3D-EVs (blue circles) were then reapplied back to the “ < 100 kDa Filtrate” samples. The effect of ASC-CM “ < 100 kDa Filtrate” from 2D (*silhouette*) and 3D (*patterned*), with/without EVs, was evaluated for ability to modulate KC **B** metabolic, **C** proliferative, and **D** migratory activity. **E** Evaluation of KC morphological changes after treatment of “ < 100 kDa Filtrate” without EVs (*top row*) and with EVs (*bottom row*) [Scale bar = 100 µm]. Significance denoted as *p < 0.05, **p < 0.01, ***p < 0.001, and ****p < 0.0001, n = 4

To isolate and observe the role of ASC-derived EV/exosomes in modulating KC re-epithelialization activity, ASC-CM was separated into > 100 kDa (EV containing) and < 100 kDa (Filtrate) fractions. Subsequently, EVs were extracted from the > 100 kDa fraction to separate them from soluble protein within the > 100 kDa fraction and used to dose 2D and 3D ASC-CM “Filtrate” fractions (Fig. 4A). Both 2D and 3D ASC-CM “Filtrate” had similar effects on KC metabolic (Fig. 4B), proliferative (Fig. 4C), and migratory (Fig. 4D) activity. Addition of 2D-EVs resulted in a slight increase in metabolic activity and no effect on proliferative activity of KCs, but 3D-EVs resulted in a significant increase for both, relative to the “Filtrate” (Fig. 4B, C). Notably, the migratory activity of KCs was significantly enhanced when ASC-CM filtrate was treated with 3D-EVs, whereas 2D-EVs significantly decreased the migratory capacity of the ASC-CM filtrate (Fig. 4D).

When comparing the morphology of KCs treated with either “Filtrate” or “Filtrate + EVs”, KCs demonstrated morphological changes seen previously. KCs treated with ASC-CM “Filtrate” had minimal morphological alterations, but addition of ASC-EVs from 2D or 3D resulted in a similar morphology to KCs seen previously after treatment with “Full” or “100 kDa” fractions (Fig. 4E).

### 3D-EVs enhance expression of basal and suprabasal cytokeratins in a dose-dependent manner

**Fig. 5:**
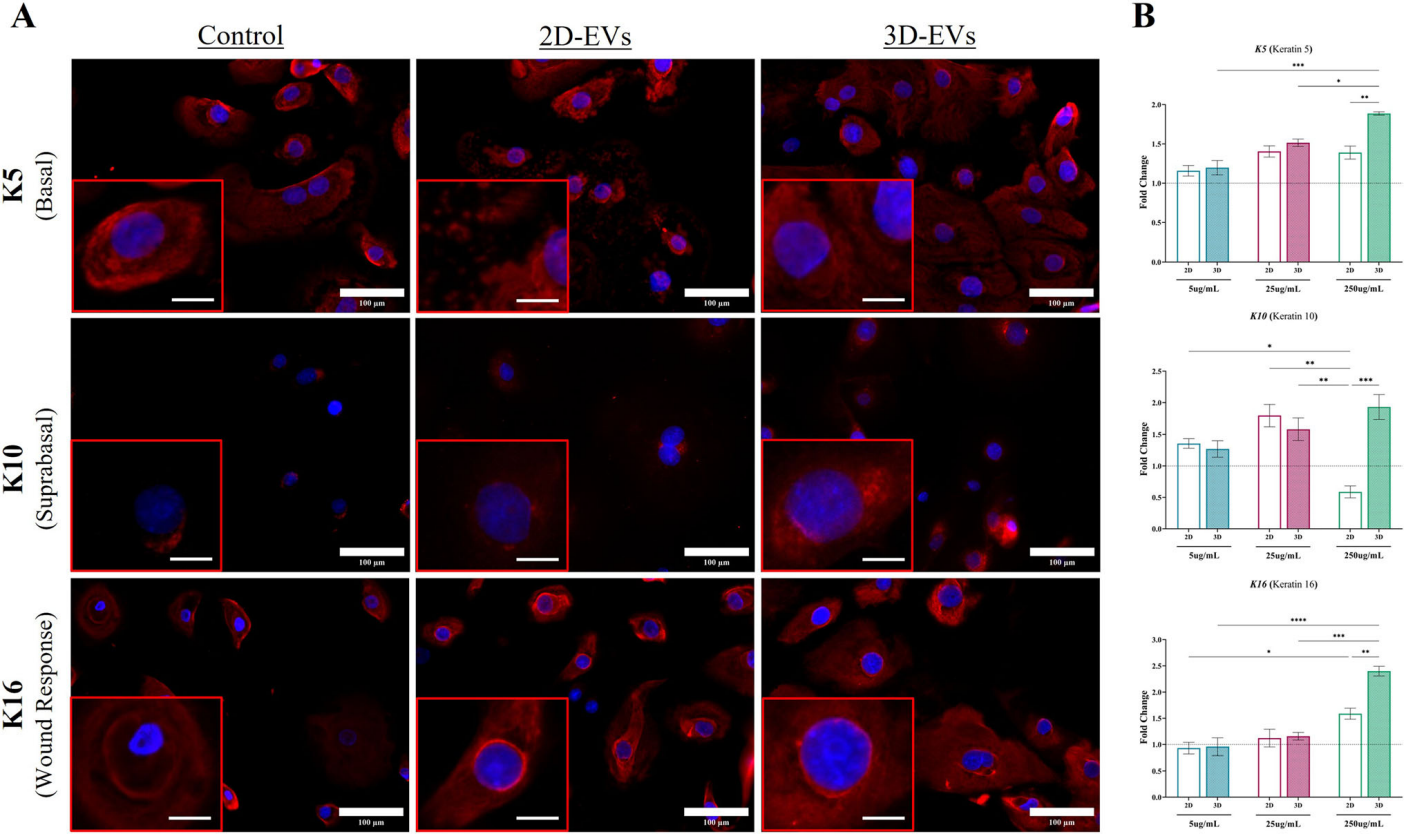
3D-EVs enhance expression of basal and suprabasal cytokeratins in a dose-dependent manner. **A** Representative images of KCs stained for cytokeratins (Red), including Keratin 5 (*top row*), Keratin 10 (*middle row*), and Keratin 16 (*bottom row*). KCs treated with KC-GM served as a control for baseline expression (*left column*), and 2D-EVs (*middle column*) and 3D-EVs (*right column*) at the highest dose of 250 µg/mL were the treatment groups [Scale bar = 100 µm, Inset Scale bar = 20 µm]. **B** ASC-EVs treatment of KCs at different doses from 2D (*silhouette*) and 3D (*patterned*) were evaluated via qRT-PCR analysis of *K5*, *K10*, and *K16*. *GAPDH* was used as an internal control. Values are represented as relative fold change to baseline control KCs indicated by dashed line. Significance denoted as *p < 0.05, **p < 0.01, ***p < 0.001, and ****p < 0.0001, n = 3

An EV dosing at 5-, 25-, and 250-µg/mL was performed to further understand ASC-EV functionality/quality and evaluate whether KC phenotype responded in any dose-dependent manner via immunolabeling (Fig. 5A) and qRT-PCR (Fig. 5B). Immunolabeling indicated that KCs treated with ASC-EVs exhibited varying degrees of protein for K5 (basal layer), K10 (suprabasal layer), and K16 (wound healing responsive) cytokeratins. Only K16 exhibited increased expression levels when treated with the highest dose (250-µg/mL) of 2D-EVs, with K10 significantly decreasing after 2D-EV treatment. Treatment with the highest dose of 3D-EVs demonstrated the capacity to significantly increase expression of all three cytokeratins at a protein and RNA level (Fig. 5A, B). Additionally, only K16 exhibited a dose-dependent response to 2D-EVs, whereas K5, K10, and K16 all exhibited a dose–response to 3D-EVs (Fig. 5B).

### EMT-like and epidermal regeneration of KCs exhibit dose-dependent response to ASC-EVs

**Fig. 6:**
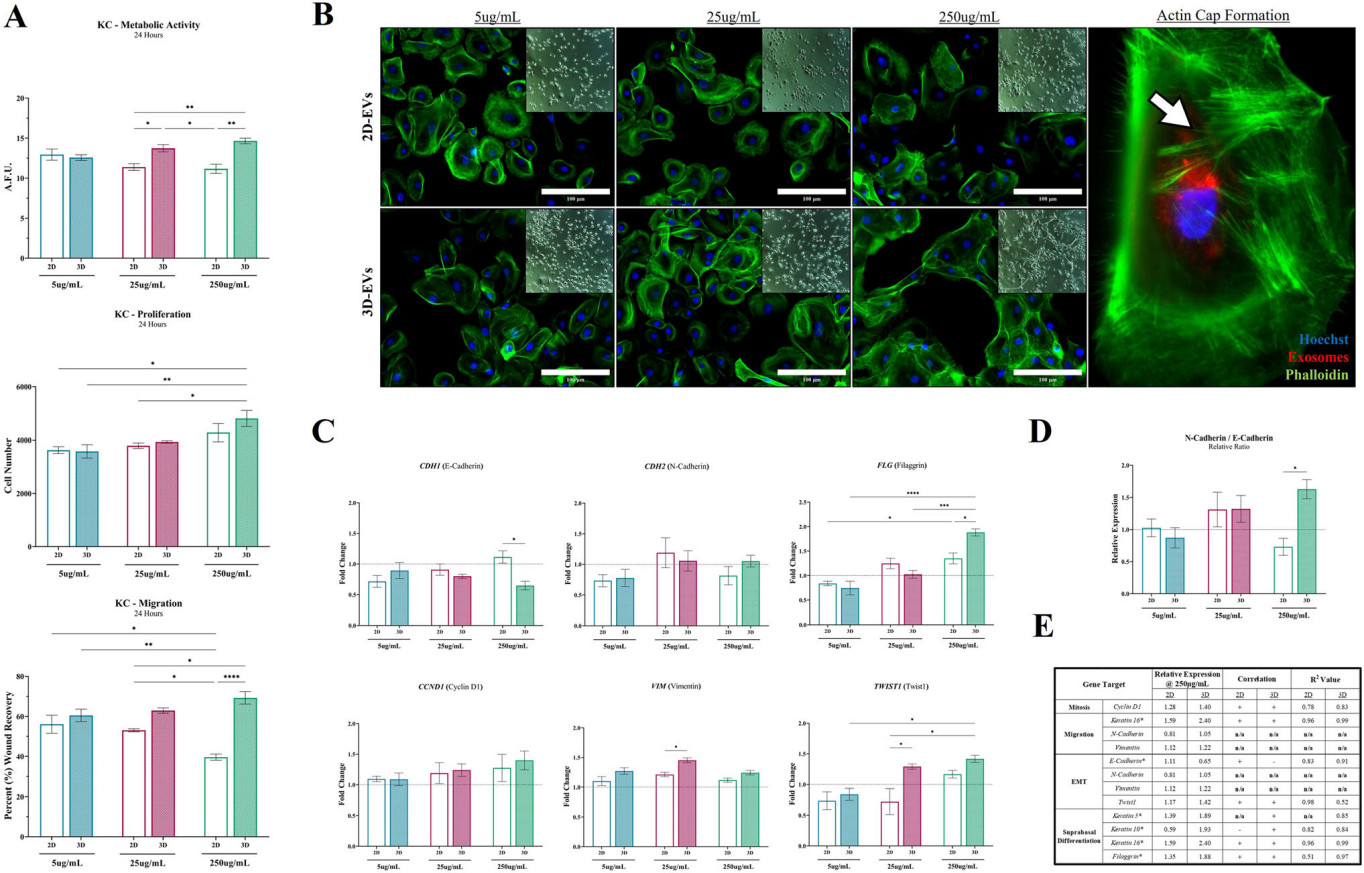
EMT and epidermal regeneration of KCs exhibit dose-dependent response to ASC-EVs. **A** The effect of ASC-EVs at different doses from 2D (*silhouette*) and 3D (*patterned*) was evaluated for ability to modulate KC metabolic (*top row*), proliferative (*middle row*), and migratory activity (*bottom row*). **B** Representative images of KC morphology and cytoskeletal changes after ASC-EV treatment. Inset is a brightfield image. Stains include Hoechst (Blue), Phalloidin (Green), DiI-labeled exosomes (Red). White arrow denotes formation of actin cap (green phalloidin). [Scale bar = 100 µm]. **C** qRT-PCR analysis of *CCND1*, *VIM*, *FLG*, *TWIST1*, *CDH1*, and *CDH2*. *GAPDH* was used as an internal control. Values are represented as relative fold change to baseline control KCs indicated by dashed line. **D** The relative ratio for RNA expression of N-Cadherin (*CDH2*) to E-Cadherin (*CDH1*). **E** Tabulated values for all genes evaluated by qRT-PCR and their respective R^2^ values and relative directionality of correlation. Significance denoted as *p < 0.05, **p < 0.01, ***p < 0.001, and ****p < 0.0001, n = 3

KC metabolic and migratory activity exhibited a decreasing trend when treated with 2D-EVs (R^2^ = 0.42 and 0.98, respectively) but an increasing trend when treated with 3D-EVs (R^2^ = 0.76 and 0.96, respectively); whereas KC proliferative activity exhibited a positive dose response for both 2D-EVs (R^2^ = 0.97) and 3D-EVs (R^2^ = 0.96) (Fig. 6A). Notably, KC morphological changes exhibited a dose-dependent response, where KCs exhibited increased spindle-like transformations, cell clustering, cell sheet formation, and cytoskeletal modulation with actin-cap formation (Fig. 6B and S5). Notably, morphological changes were observed in both 2D-EVs and 3D-EVs, but more pronounced with 3D-EVs treatment at equivocal doses.

Additionally, a number of key gene markers for KC wound healing activity and phenotype were assessed to determine whether KC gene expression exhibited similar changes as the functional and proteomic evaluations (Fig. 6C). The expression of the key junctional/migratory markers *CDH1* (E-Cadherin) exhibited a dose response to ASC-EVs, whereas *CDH2* (N-Cadherin) did not demonstrate any significant dose response. Additionally, the highest dose of 3D-EVs resulted in a significant decrease in expression of CDH1 (relative to 2D-EVs). Whereas the expression of the cell cycle proliferative marker *CCND1* (Cyclin D1) did demonstrate a dose response with increasing ASC-EVs concentration from both 2D and 3D (Fig. 6C). Similarly, expression of *FLG* (Filaggrin), a marker for suprabasal differentiation (granular layer), exhibited a dose response to ASC-EVs, with 3D-EVs at 250-µg/mL providing a significant increase relative to 2D-EVs. Whereas *VIM* (Vimentin), a marker for KC migration, did not exhibit a dose response (Fig. 6C). *TWIST1*, a marker for epithelialization and an EMT-like response, exhibited a dose response to ASC-EVs with a more potent effect from 3D-EVs (Fig. 6C). When considering the relative expression of both *CDH1* and *CDH2*, KCs exhibited a significantly increasing trend towards favoring expression of CDH2 (N-Cadherin) (Fig. 6D). The relative correlation of each gene expression marker is denoted in Fig. 6E.

## Discussion

The diverse array of tissue types and cell populations involved in wound healing, in addition to the wide-ranging patient populations and various types of wounds, highlights the need for therapies that are tailored for specific applications. Recently, investigations into the utilization of the dynamic secretome of MSCs has demonstrated the potential for a unique opportunity for advancing the field of regenerative medicine and personalized wound healing modalities [14, 39, 40]. To date, the secretome of MSC-like populations have been shown to stimulate a variety of wound healing functions, including modulation of fibrotic activity in fibroblasts [41], promotion of angiogenic activity in endothelial cells [42], and increased keratinocyte migratory activity [43].

Although preliminary data with MSC biologics are promising, in order to tailor regenerative biologic therapies more effectively there is need to differentiate the role of MSC secretome components and generate modalities to reproducibly create efficacious products. Previous studies have begun investigating the utility of a number of MSC secretome elements, including looking at the effect of a single component, such as production of a specific growth factor (e.g., VEGF) or exosomes [44–46], whereas other studies have focused on the effect of complete or non-filtered conditioned media from MSCs [47, 48]. Ultimately, the ideal composition of factors will be dependent on the application, though the diverse milieu of factors secreted by MSCs offers a dynamic balance of factors that can be applied to a variety of clinical scenarios.

However, to date, most research into MSC biologics utilize 2D culture systems, and the ones that do use 3D systems focus on using systems for cell delivery and/or production of a specific secretory product via a “priming stimulus” (e.g., fibrin hydrogels enhance VEGF secretion) [49]. Thus, current data are incomplete and obstructed due to the detrimental effects of traditional 2D systems on MSC phenotype, viability, and regenerative capabilities [50, 51]. The unphysiological environment of 2D systems drastically hinders the standardization and reproducibility of MSC-based therapies. Similarly, many current 3D systems (e.g., spheroids and microcarriers), although more efficacious than 2D for a variety of applications, still lack tissue-mimetic properties and/or have diffusional constraints that result in cellular heterogeneity and hinder large-scale expansion efficiency that would be necessary for future clinical therapies [52, 53].

Therefore, this study utilized a tissue-mimetic 3D hydrogel system that has previously shown to enhance the retainment of non-senescent, MSC-like populations, relative to standard 2D culture [31]. When designing a tissue-mimetic system, there are a number of important mechanical properties of tissue to consider, including the compressive, storage, and loss moduli, which are denoted in Table 1 for our hydrogel system as it compares to previously published data on adipose tissue [28, 54, 55].

Two key benefits of this system are that it allows the easy collection of secreted byproducts due to unique architectural design. Unlike previously investigated poured/molded 3D hydrogels in literature that lack defined microchannels and structure, the micro- and macro-architecture provides a unique opportunity for continuous expansion of cells within a hydrogel system while permitting the easy and continuous collection of secreted byproducts, such as EVs. The microchannels help circumvent diffusional limitations that are associated with traditional hydrogel systems, subsequently increasing the efficiency of secreted biologics collection. The other key benefit of this system is how the tissue-mimetic environment results in MSC-like populations that retain their adaptive and regenerative capabilities, allowing them to respond to stimuli more appropriately, like they would *in vivo*. As previously demonstrated with this unique hydrogel system [31, 37], the improved ASC phenotype within this adipose-like microenvironment results in a secretome composition that enhances regenerative responses, relative to 2D. In part, this is likely due to a combination of increased secretion of anti-regenerative factors from 2D cultured cells, such as ROS and senescence-promoting factors (Fig. 2F), in addition to the increased secretion of pro-regenerative factors from 3D cells.

The ability to achieve targeted and rapid degradation of the hydrogel substrate allowed easy extraction of ASCs for RNA analysis. To support and highlight the relative benefit of our 3D system on MSC phenotype, an RNA array was performed and demonstrated that ASCs exhibited a significant retainment of key MSC and MSC-like markers in 3D, relative to 2D (Fig. 1C and Table S1). As we can see, surface markers associated with an MSC-like phenotype, including *NT5E* (CD73), *THY1* (CD90), *ENG* (CD105), *ALCAM* (CD166), and *NGFR* (CD271), retain a gene expression profile similar to the primary (P1) ASC populations within the 3D hydrogel, whereas the 2D system resulted in a precipitous decline in their expression. Moreover, ASCs in 3D exhibited retained or increased expression of several “stemness” associated markers, including *LIF*, *OCT4*, *HGF*, *ZFP42*, and *NOTCH1*, in addition to increased retainment of multipotency markers *PPARG* (adipogenic), *RUNX2* (osteogenic), and *SOX9* (chondrogenic). Notably, there is a significant decrease in *CASP3* expression in 3D-ASCs, an important marker for cell health and induction of cell death via apoptosis.

Even though the same seeding density was utilized initially, variations in cellular proliferation were expected, which can be seen by when looking at the relative proliferative activity via the population doubling time (PDT) of ASCs extracted from both the 2D and 3D culture systems (Fig. 1F). Therefore, cell numbers were assessed in advance for 2D and 3D to determine the optimal days for collection of ASC-CM to standardize relative media-per-cell ratios. Additionally, both 2D and 3D ASCs were at P2 upon seeding and remained in culture for 8 days for this study. Thus, any differences noted reflect the culture environment and not subculturing effects. Based on prior literature, collection of ASC-CM in 2D at 60–80% confluency was desired.

Previous studies have demonstrated a positive impact of the MSC secretome on KC wound healing activity [43, 56]. However, to date, the data are limited and there are mixed results on what components may be driving specific activities, such as migration and proliferation, due to the adaptive and dynamic nature of the secretome. Therefore, KC functional and phenotypic assessments were performed as an indirect surrogate measure and qualification of MSC secretome components from either a 2D or 3D culture system. Molecular weight stratification was utilized due to the minimally invasive nature of this methodology that does not require solvents or other factors to precipitate and/or isolate proteins from conditioned media, in addition to the ability to compartmentalize compounds into predefined fractions and standardize a consistent processing methodology. Notably, ASCs cultured within our 3D culture system exhibited enhanced secretion of proteinaceous compounds, relative to 2D (Fig. 2B). Upon stratification, only the “100 kDa” fraction was significantly increased in 3D, relative to the 2D counterpart. This suggests that the enhanced protein production and regenerative capacity of the 3D ASC-CM is likely, in part, a result of the “100 kDa” fraction, which consists primarily of larger growth factors/cytokines, matrix-related proteins, and EVs.

KC functional activity was then evaluated to assess ASC-CM secretome activity for each MW fraction. The functional benefits of ASC-CM from the “100 kDa” fraction demonstrated the greatest positive impact on KC activity and paralleled the effect and benefits seen in the “Full” media group, including KC morphological, metabolic, proliferative, and migratory activity. Whereas the lower MW fractions had less effect on KC functional activity, a closer look demonstrated that the effect of the lower MW fraction from both 2D and 3D are similar but there is a significant drop-off in effect from the 3D “100 kDa” fraction (Fig. 2G, I). This is potentially due to the increased benefits from the 3D “100 kDa” fraction, rather than a negative effect of lower MW 3D compounds. Interestingly, both 2D and 3D “Full” and “100 kDa” groups induced significant morphological changes in KC populations that resembled more flattened, stratified, and spindle-shaped cells forming cellular sheets. Whereas lower MW fractions had minimal effects on the KC morphology. Moreover, further evaluation of KC nuclear size indicated an increase in nuclear surface area in the “Full” and “100 kDa” groups only (Fig. S3), potentially due to flattening of the cells seen in the morphological changes, resulting in a decrease in the cellular z-axis. Future studies will investigate the relationship between KC morphological changes and wound healing activity; however, some KCs are thought to undergo a transitional form similar to that of an epithelial-to-mesenchymal transition (EMT), in addition to suprabasalar differentiation during re-epithelialization, which could both account for the morphological changes seen [5, 57, 58].

Previous studies have shown that senescent cell populations, including MSCs, can impair the regenerative activity of surrounding cell populations in a number of ways, including the secretion of factors that induce senescence [59]. Notably, we have previously shown that ASCs cultured within this tissue-mimetic system delays the induction of ASC senescence, whereas 2D culture led to a rapid induction of senescence [31]. Therefore, 2D cultured ASCs may be secreting protein or non-protein factors associated with senescence and decreased cell health that are then inducing senescence in KCs (Fig. 1F and S4). Analysis of KCs after ASC-CM treatment indicated that the “10–3 kDa” MW fraction from 2D did have a greater propensity for inducing senescence in KC populations, with the 2D “30–10 kDa” fraction also demonstrating an increasing trend. Interestingly, the analogous fraction in 3D did not induce senescence. Since the relative amount of protein secreted in the lower MW fractions was similar between 2D and 3D, the senescence induction is not a result of protein quantity but rather due to either the protein quality/composition or non-proteinaceous factors, such as reactive oxygen species (ROS). Notably, impaired balance of a number of secretory factors is considered to be able to promote the progression of cellular senescence in a paracrine manner. Moreover, the senescence-associated secretory phenotype (SASP) of MSC-like populations has previously been shown to induce senescence in other cells, potentially via ROS or inflammatory factors within the 10–30 kDa range. However, the dynamic nature of the SASP warrants further investigations into the exact mechanisms of senescence induction.

To further investigate the spindle-like morphology change and enhanced migratory activity of KCs treated with high MW ASC-CM fractions, Vimentin was assessed and found to be correlated to KC migratory activity. Vimentin was significantly increased in KCs treated with the “100 kDa” and “100–30 kDa” fractions in 3D, relative to the lower MW fractions, especially at the outer edges of cell sheets. However, only the “100–30 kDa” fraction was enhanced in 2D, with the “100 kDa” fraction in 2D demonstrating a reduction in capacity to promote KC migration and express Vimentin. This indicated that components within the “100 kDa” fraction are likely promoting KC migratory activity within the 3D ASC-CM and secretion of these factors is decreased/countered within 2D culture. Moreover, there is likely a secondary set of factors within the “100–30 kDa” fraction that is also promoting migratory activity of KCs that is similar between 2D and 3D. These data suggest that the “100 kDa” and “100–30 kDa” fractions of the ASC secretome contain compounds that modulate KC migratory activity. Thus, culture of ASCs within a tissue-mimetic system either enhances the secretion of positive “100 kDa” factors or 2D culture promotes the secretion of negative “100 kDa” factors, but does not alter the migratory factors within the”100–30 kDa” range. This is the first demonstration of the diverging effects of secretome fractions separated via MW and provides insight into the functional implications of specific biomodulatory fractions. In this case, the regenerative migratory stimulus within the “100 kDa” range can be significantly modulated via culture conditions, with an apparent “inversion” in activity and negative stimulus in 2D culture conditions. Additionally, previous studies have demonstrated that increased expression of vimentin at the leading migratory edge of the epidermis may be a critical step to epithelial regeneration natively [60–62]. Excitingly, our data depict that treatment with the “100 kDa” fraction of the ASC secretome can promote this physiological KC wound healing response.

Based on the observed benefits of the “100 kDa” fraction, further stratification of the “100 kDa” components was carried out to investigate whether EVs within the “100 kDa” fraction were driving the observed functional changes in KCs. Culture of ASCs within the tissue-mimetic system resulted in increased production of EV particles, including modulation of the relative proportion of EV-to-Secreted protein, indicating that the secretome dynamics of ASCs tend to favor secretion of EV particles rather than soluble protein when in a more tissue-mimetic, native environment. Moreover, the relative size distribution of the EVs from ASCs in 3D trended towards a slightly smaller size, potentially suggesting that EVs are more likely exosomal in nature (25–250 nm) rather than MVs (100–1000 nm) or apoptotic bodies (200–2000 nm). To the authors’ knowledge, this is the first demonstration of a shift in MSC secretory dynamics towards favoring the secretion of EVs from an MSC-like population within a tissue-mimetic system rather than soluble protein (Fig. 4B), providing potential insight into native regenerative tissue signaling preferences. Whereas previous studies have only investigated total EV amount or effect in 3D systems without comparing to 2D or controlling for total relative secreted protein.

The role of the EVs from both 2D and 3D were then evaluated for ability to recover and improve KC functional wound healing activity by utilizing < 100 kDa filtrate dosed with or without ASC-EVs to determine whether the benefits seen from the “Full” and “100 kDa” fractions are an effect of EVs. Since EVs are primarily located within the “100 kDa” fraction, the “ < 100 kDa Filtrate” groups plus ASC-EVs resemble “Full” media, from the stratification studies, but depleted of soluble “100 kDa” protein that is not derived from EVs. The previous MW stratification data suggested that the “100 kDa” fraction provided minimal change in KC metabolic and proliferative activity, relative to the “Full” group, for both 2D and 3D ASC-CM. When separating out the EVs and dosing the “ < 100 kDa Filtrate”, we see that both 2D-EVs and 3D-EVs are able to improve KC metabolic activity. On the other hand, only dosing with 3D-EVs was able to significantly increase the effects of the “ < 100 kDa Filtrate” when it came to KC proliferative activity. Notably, KCs did exhibit a significant increase in proliferative activity in the “2D < 100 kDa Filtrate + 3D-EVs” group, suggesting that the contents within 2D-EVs may lack the same proliferative capacity as 3D-EV contents. Thus, the previous proliferative benefits seen in the “100 kDa” fraction for 2D may be non-EV derived or the EV stimulus is less robust.

Furthermore, comparing the effect of 2D-EVs to 3D-EVs on KC migratory activity reveals a significant differential. As we saw in the “100 kDa” fractions previously, there is an “inversion” of migratory signaling between 2D-EVs and 3D-EVs, where 3D-EVs enhance KC migration but 2D-EVs hinder KC migratory activity. These data support the stratification data that suggested that “100 kDa” fraction of 2D ASCs does not contain the same pro-migratory stimulus as their 3D counterpart, rather 2D-EVs may contain a possible anti-migratory stimulus for KCs. One possible explanation for this may be that the adaptive response of ASCs in 2D monolayers to the overcrowding and contact inhibition resulting in ASCs releasing anti-migratory (and possibly anti-proliferative) signals within EVs. Conversely, 3D culture results in more regenerative ASC populations and provides significantly more surface area to migrate which likely promotes pro-migratory and pro-proliferative signaling between cells. Notably, the effect of the "< 100 kDa Filtrate” from both 2D and 3D have comparable effects on the KC functional activities. This suggests that the differential in activity after “Full” 3D ASC-CM treatment is likely a result of the “100 kDa” fraction and that there is likely minimal differences between the “< 100 kDa Filtrate” from 2D and 3D, which is further supported by the change in secretory activity seen in Fig. 2B.

Previous studies have demonstrated how the contents of EVs can change depending on the cell type, tissue source, or donor, however; the culture conditions are often not taken into consideration. In this study, EVs derived from the exact same tissue and donor have a shift in regenerative potency when collected from ASCs from a tissue-mimetic culture system. This suggests that the ability to retain the more robust and regenerative phenotype of primary ASCs likely results in retainment of ASCs with pro-regenerative properties, where subsequent production of EVs and their contents can drive wound healing processes, such as epithelialization. Whereas in traditional 2D culture, a precipitous decline in ASC phenotypic properties ensues and the potency of EVs declines, which is highlighted by the EV dosing experiments within this study. So not only has the quantity of EVs produced in 3D increased, as previously described, but the quality has improved.

Interestingly, a closer look at the stratification and EV analyses reveal that the morphological changes exhibited by KCs after treatment with “Full” or “100 kDa” ASC-CM are potentially independent of KC migratory capacity but are reliant on the ASC-EV fraction. This can be seen when comparing the similar relative migration of KCs after treatment with the “100 kDa” and “100–30 kDa” fractions from 3D, but only the “100 kDa” induced morphological changes. Moreover, the “ < 100 Filtrate” fraction is still unable to dramatically alter KC morphology even though it contains the “100–30 kDa” migratory stimulus, but addition of 2D-EVs or 3D-EVs results in the same morphological changes seen within the “100 kDa” fraction (Fig. 4E). It is also important to note that both 2D-EVs and 3D-EVs induce KC morphological changes to varying degrees, even though 2D-EVs appear to contain an anti-migratory stimulus. Similarly, the reliance of KC morphology changes on ASC-EVs is further supported by the DiI-labeling and *in vitro* tracking data of ASC-EVs, where the KCs undergoing the most drastic morphology changes are the ones taking up the greatest proportion of EVs (Fig. S5).

Lastly, an EV dosing study was performed to further understand ASC-EV functionality at comparable doses and if KCs exhibited any dose-dependent wound healing responses to the EVs and/or changes in KC phenotype. Native expression of K5 (and K14) is typically considered to decline during wound healing and K16 (and K6) are considered to increase, with K10 more of an indicator of suprabasal differentiated KCs that can denote granular layer KCs when paired with increased expression of FLG [5]. Suprabasal K10 expressing KCs are considered to switch to K16/K6 expression during the wound healing process. K16/K6 expression is thought to play a critical role in collective cellular migration of KCs [5, 63]. In this study, 3D-EVs appear to significantly enhance expression of all cytokeratin markers in a dose-dependent manner. Whereas 2D-EVs led to a decline in K10 expression and formation of sporadic aggregates of K5 versus the more homogenous cellular distribution seen in 3D-EV treated KC, possibly hinting at enhanced K5 protein turnover and remodeling occurring in 2D-EV treated KCs.

Further assessment with functional and RNA analysis demonstrated a positive dose-dependent correlation of KC metabolic, proliferative, and migratory activity when treated with 3D-EVs. Whereas treatment with 2D-EVs contained a negative correlation with KC migratory activity and a positive correlation in proliferative activity. These data further demonstrate the anti-migratory stimulus for KCs found within ASC-EVs from 2D culture. Both 2D-EVs and 3D-EVs were able to promote a dose-dependent morphology change in KCs; however, 3D-EVs contained a more potent stimulus with the effects of the low dose 3D-EVs (5-µg/mL) mirroring that of the high dose 2D-EVs (250-µg/mL). Since it is unlikely that a single given cell is undergoing migration, proliferation, and differentiation all at the same time, the more likely scenario is that a higher proportion of the untreated keratinocytes are originally not actively undergoing these processes and instead are delegating metabolic activity to other process or cell maintenance. However, upon addition of the ASC-EVs, the keratinocytes subsequently shift their metabolic activity towards increasing migration, proliferation, or differentiation, with the stimulus from 3D-EVs being more potent. Therefore, keratinocytes originally only had a fraction of the cells actively undergoing one of those activities, but a much higher proportion of cells were prompted to undergo migration, proliferation, or differentiation after EV addition. This concept is similar to what is hypothesized to occur natively during wound healing, where upon injury, keratinocytes undergo a phenotypic switch resulting in a leading migratory tongue of keratinocytes that “pulls” along the cell sheet with back-filling of more proliferative keratinocytes [64, 65].

It is important to note the key morphological features changing within KCs after ASC-EV treatment that appear to happen in a stepwise manner. First, KCs begin to cluster together and form small satellite-like colonies that eventually merge into bigger colonies. Second, KCs begin to alter their cellular morphology from a rounded shape to a more irregular spindle-like shape, with rearranged cytoskeletal structures and increased cellular extensions. Lastly, KCs begin to firmly attach and integrate with one another to form a continuous cellular sheet with more flattened cellular populations. The correlation between EV/exosome uptake and formation of a nuclear actin-cap within the KC cell sheets was an interesting and unexpected finding which likely played a direct role in the morphological flattening and increased nuclear surface area discussed previously. Moreover, actin-cap formation promotes nuclear flattening and is a known modulator of gene expression and is often associated with mechanotransducive and epigenetics responses [66]. Broadly speaking, both 2D-EVs and 3D-EVs were able to induce this KC response to varying potencies. However, where 3D-EVs diverge is their enhanced capacity to stimulate migratory activity in KCs. This is the first demonstration of diverging migratory activity of ASC-EVs and its possible association with KC morphological changes. To the authors’ knowledge, this is the first *in vitro* demonstration of substantial EV-induced KC morphological changes (e.g., actin-cap and migratory cell sheet formation) and its potential association with KC wound healing activity; thus, EV-induced KC morphological changes requires further investigation.

Interestingly, the RNA profile of the KCs treated with ASC-EVs demonstrated a diverse phenotypic profile. Both 2D-EVs and 3D-EVs demonstrated a dose-dependent response in several key KC genes, including *K10, K16, FLG, TWIST1*, and *CCND1*. However, the augmentation of KC wound healing activity was more pronounced at a functional and RNA level after 3D-EV treatment compared to 2D-EVs. Notably, 3D-EVs were able to promote increased expression of a proliferative and basal-like phenotype (*CCND1* and *K5*), a differentiated suprabasal-like phenotype (*K10* and *FLG*), a wound healing phenotype (*K16*), and a more migratory phenotype (*K16* and *TWIST1*). Moreover, KCs treated with 2D-EVs demonstrated apparent breakdown and turnover of K5 protein (Fig. 6A), suggesting a possible transition period where the KCs were losing their basal-like phenotype. A closer look also demonstrates that 3D-EV treated KCs exhibit an EMT-like transition with increased expression of *TWIST1* and an increased N-Cadherin-to-E-Cadherin ratio, which is further supported when paired with the increased vimentin production, morphological changes, and enhanced migration. Together, these data suggest that ASC-EVs from 3D are able to more effectively stimulate KCs to form a stratified collective cell sheet, consisting of EMT-like migratory KCs forming the leading edge of the cell sheets, while maintaining cell-to-cell adhesion in the interior of the cell sheet and expression of cytokeratins associated with suprabasal differentiated (K10), wound responsive and migratory (K16), and proliferative (K5) phenotypes.

Notably, collective cell sheet migration is a proposed mechanism of native KC re-epithelialization and to date, this process has been poorly recapitulated *in vitro* [65, 67]. It is thought that 2D monolayer cultures of KCs are unable to recapitulate the proper differentiation and morphological changes necessary to promote stratification and collective cell sheet migratory activity of KCs [67, 68]. However, our data suggest that EVs may in fact be a critical link and key in promoting this native regenerative process that is considered to occur *in vivo*. Previous studies utilizing traditional culture expansion for EV collection have demonstrated the ability to translate to improved tissue regeneration activity *in vivo*, with a number of different *in vivo* models demonstrating benefit after treatment with MSC-derived EV/Exosomes [69–73]. This suggests that EV/Exosomes may in fact be a critical component to native regeneration for a variety of tissue types, and that the augmentation capabilities of EVs in tissue regeneration may be further enhanced with a tissue-mimetic culture system for secretory biologics production and collection, as demonstrated in this study. To the authors’ knowledge, this is the first demonstration of the ability of ASC-EVs to broadly promote multiple key functional phenotypic changes in KCs rather than just migration and/or proliferation, and provides new insight into potential native *in vivo* signaling dynamics of wound repair that warrants further investigations. More specifically, the apparent capacity for KCs to retain expression of key basal and suprabasal epithelial-like cytokeratin markers simultaneously, while also expressing mesenchymal-like markers (*VIM, CHD2, TWIST1*) after EV treatment warrant further study.

There are a few components of this study that require further investigation that are limiting in their current form but do not ultimately detract from the study findings. Although one ASC source was used, the comparative study design between 2D and 3D allowed for any donor specific effects to impact each group relatively the same. Similarly, the effects of the ASC-CM were only tested on KCs. The response of different cell populations to ASC secretome components, such as exosomes, could be dependent on the secondary cell populations (e.g., fibroblasts) and thus further studies with other cell populations is warranted to establish a more holistic idea as to what secretome formulations are best for specific applications/responses. Moreover, the utilization of only *in vitro* analyses can be limiting, however, the utilization of a wide-range of functional, proteomic, and RNA assays provided a holistic perspective that provided data that can be used for future *in vivo* study designs and provides the framework for stratifying the effects of secretome components. Additionally, although EV/exosomes appeared to drive many of the KC functions tested, soluble proteins may be important for other processes or act additively/synergistically with EV/exosomes in some situations. This study is only able to evaluate the effect of protein secreted from ASCs and thus is not inclusive of all soluble proteins; however, EVs in this study are able to demonstrate the ability to enhance several key wound healing functions in KCs. Lastly, although outside the scope of this study, future studies into the dynamic array of contents within EV/exosomes (e.g., proteins, ROS, nucleic acids, miRNA) should be further investigated to better understand how certain environmental conditions shift the contents of EVs and determine the most effective delivery modalities for specific clinical therapies. However, it remains to be determined whether culture of MSC-like populations in a tissue-mimetic environment increased the production of more regenerative factors (due to retainment of more robust MSC populations), and/or did 2D culture decrease the quality of the factors being secreted via increased secretion of anti-regenerative compounds (both soluble protein and EV-derived), or some combination of the two.

In conclusion, in this study we highlight the importance of understanding the role of culture environment on MSC-like cells, their phenotype, and their capacity to secrete regenerative compounds by performing a direct comparison of the regenerative functionality of the ASC secretome from both 2D culture and a tissue-mimetic system. It is important to note that MSC-like populations are dynamic and highly adaptive to their environments, thus the secretome and/or EV/exosomes from one population may vary depending on the system utilized to generate the biologics. Therefore, the effect of any system should be evaluated before generalizing the effects of specific MSC-derived secretome components for future translatable therapies. Additionally, we demonstrate that EV/exosomes may be a key driver of epidermal regeneration activity within the ASC secretome and, when generated within a tissue-mimetic system, are able to enhance the formation of collective, migratory cell sheets of KCs, a critical process during native epidermal regeneration. EV/exosomes offer a unique approach to improving current wound healing modalities clinically due to their enhanced stability, relative to proteinaceous biologics (growth factors), and their diverse/dynamic compositions. Thus, EV/exosomes could act as adjuvant therapies via integration into current wound dressing and/or injectable hydrogel systems in order to tailor therapies and enhance wound outcomes. Overall, this study helps provide new insight into the direct role of EV/exosomes on KC wound healing and epidermal regeneration and warrants further investigation of potential clinical benefits that MSC-derived EV/exosomes may provide.

### Supplementary Information

Below is the link to the electronic supplementary material.

Supplementary file1 (DOCX 2076 KB)
