# Supplementary material for: Mesenchymal Stem Cell Extracellular Vesicles from Tissue-Mimetic System Enhance Epidermal Regeneration via Formation of Migratory Cell Sheets
Source: Tissue Eng Regen Med. 2023 Jul 29;20(6):993–1013. doi: 10.1007/s13770-023-00565-6 (PMC10519905; doi:10.1007/s13770-023-00565-6)
Supplement: Supplementary file 1 — Supplementary file1 (DOCX 2076 KB) [file 13770_2023_565_MOESM1_ESM.docx]

**Supplemental Data and Figures**

**
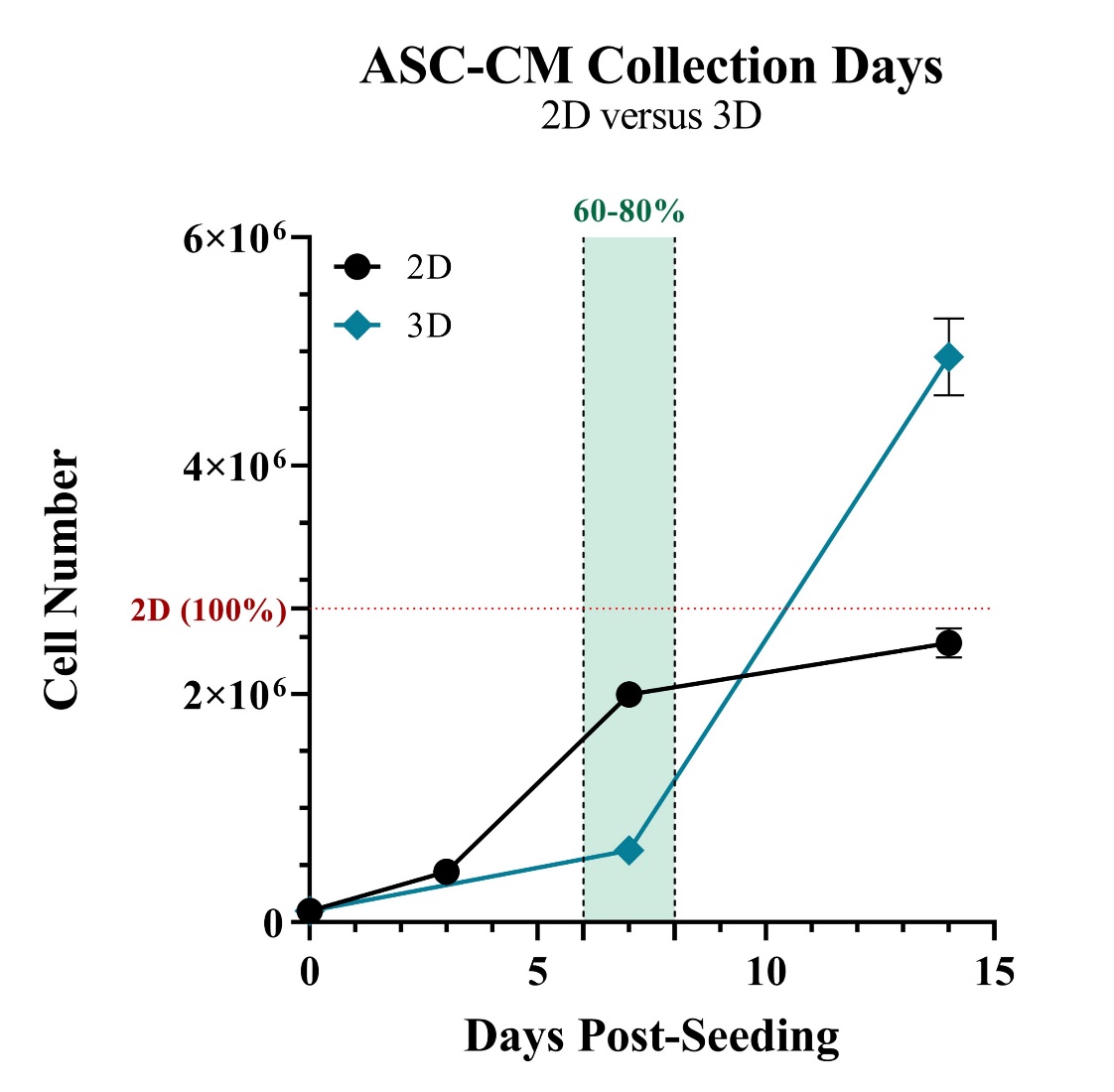
**

**Supplemental Figure 1: ASC Growth Curve for 2D and 3D to Establish ASC-CM Collection Days.** ASCs cultured in the 3D system had a slight delay in reaching the growth phase likely due to an initial migratory phase to equally distribute throughout hydrogel microarchitecture, but quickly overcame the relative cell number in 2D and eventually exhibited a higher rate of proliferation in 3D. Therefore, cell numbers were assessed in advance for 2D and 3D to determine the optimal days for collection of ASC-CM to standardize relative media-per-cell ratios. Based on prior literature, collection of ASC-CM in 2D at 60-80% confluency was desired and days 6-8 were selected to collect ASC-CM. Media volumes were adjusted accordingly to account for differences in cell numbers between 2D and 3D.

**
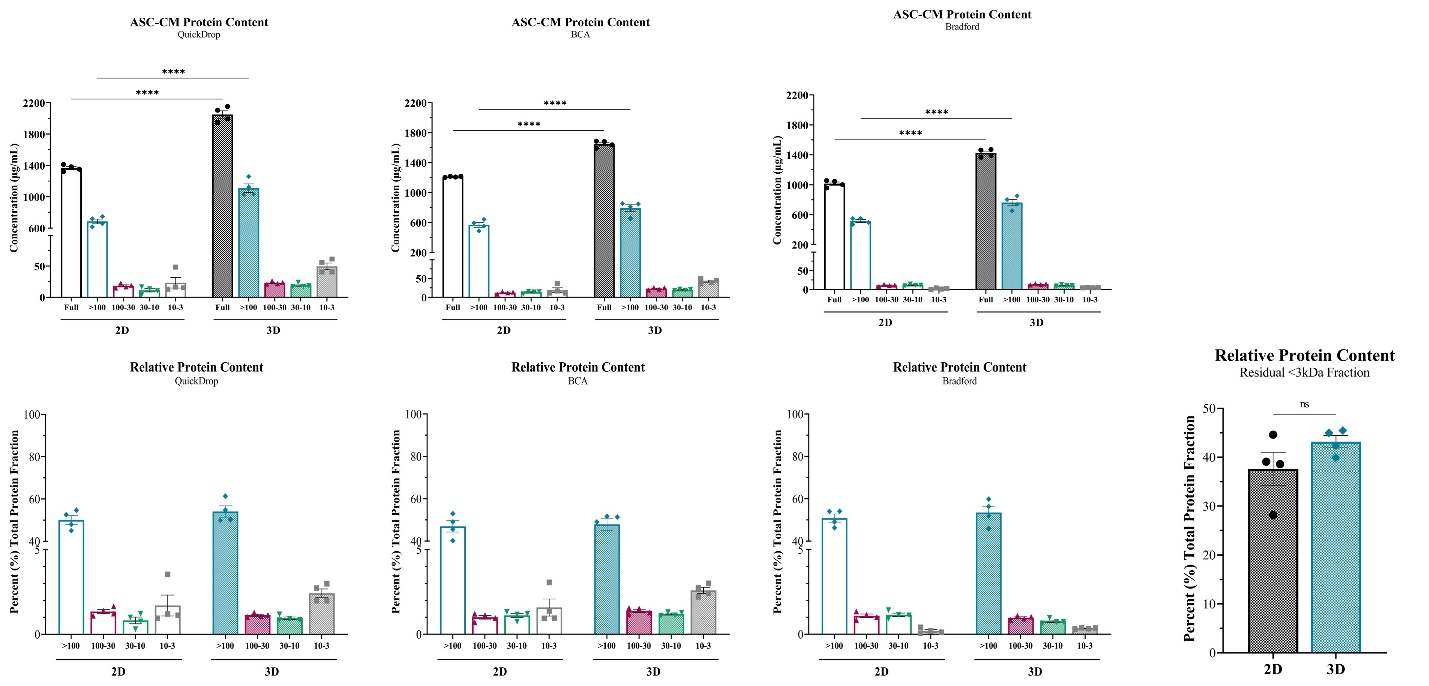
**

**Supplemental Figure 2: Quantification of ASC-CM Protein Content.** Three (3) separate protein quantification methodologies were utilized to determine EV content within ASC-CM, QuickDrop, BCA, and Bradford. All three (3) methods exhibited the same trends though absolute values slightly varied due to inherent differences in the assay principles. Absolute protein values (top row) are depicted and relative protein values (bottom row) are depicted. Additionally, residual “<3kDa” filtrate was assessed but not used in experimental assays since unable to concentrate the ASC-CM fraction in the same manner as the others. The ”<3 kDa” fraction also depicts no significant difference in content between 2D and 3D ASC-CM.

**
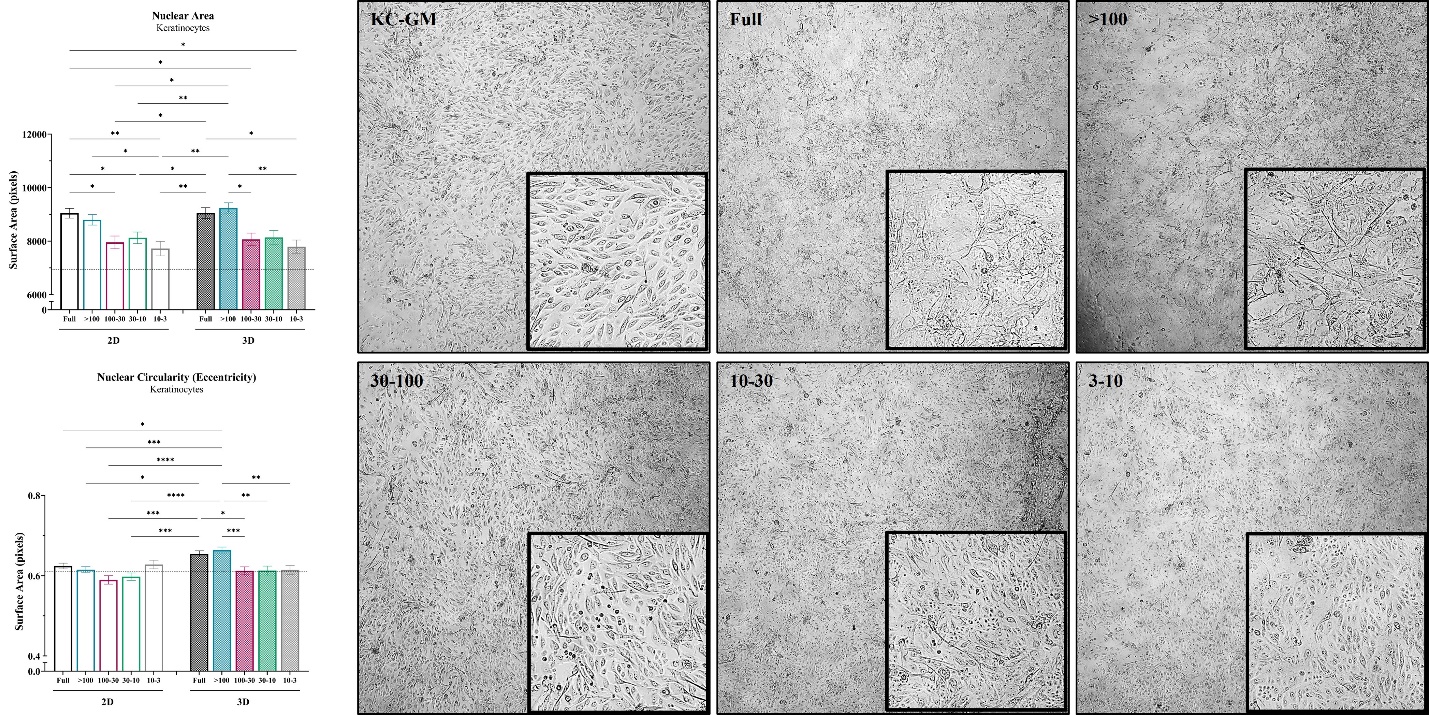
**

**Supplemental Figure 3: KC Morphological Changes after MW Stratified ASC-CM Treatment.** Representative morphology images of KCs after treatment with KC-GM supplemented with different ASC-CM MW concentrates for 24 hours. Only the “Full” and “>100 kDa” fractions induce similar KC morphological changes, including spindle-cell formation, stratification, and collective cell sheet formation. KC morphology changes were seen to varying extents for both 2D and 3D ASC-CM. Moreover, CellProfiler was used to assess nuclear area and circularity. KCs treated with “Full” or “<100 kDa” fraction exhibited nuclei with a larger surface area and less circular shape (0-1 scale with 0.5 = to a circle). Likely a result, in part, to cell flattening and consequently, nuclear flattening.

**
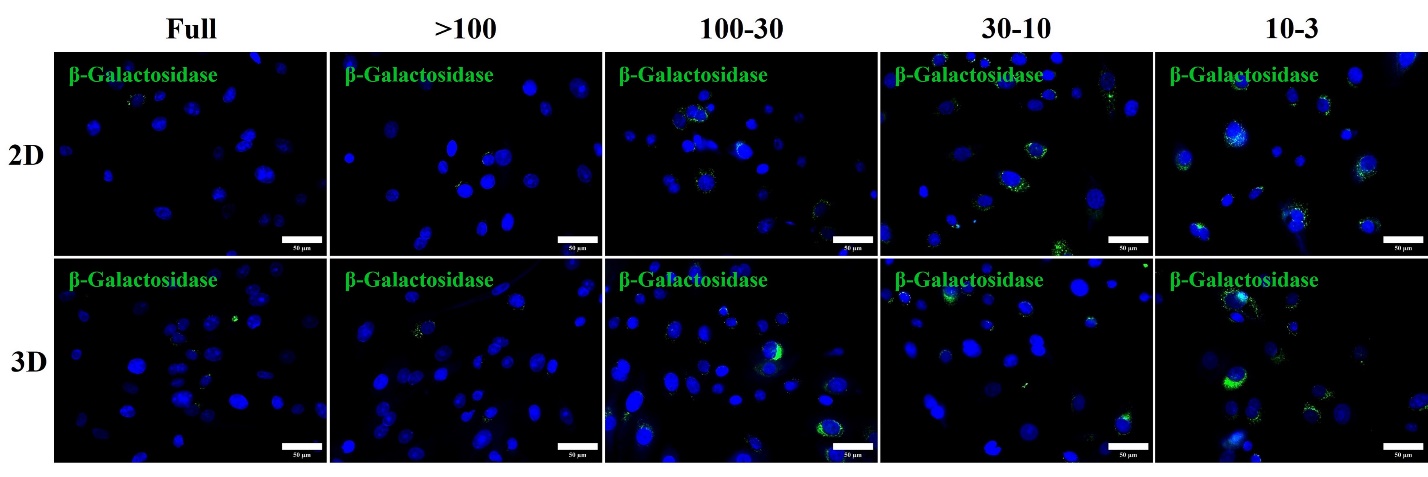
**

**Supplemental Figure 4: Image Analysis of KC Senescence after MW Stratified ASC-CM Treatment.** Representative images of KCs after treatment with KC-GM supplemented with different ASC-CM MW concentrates for 24 hours. Β-Galactosidase activity was used as a surrogate measure for senescence. Lower MW fractions of ASC-CM appeared to induce senescence in KC populations. This corroborated the qRT-PCR data for p16 previously discussed.

**
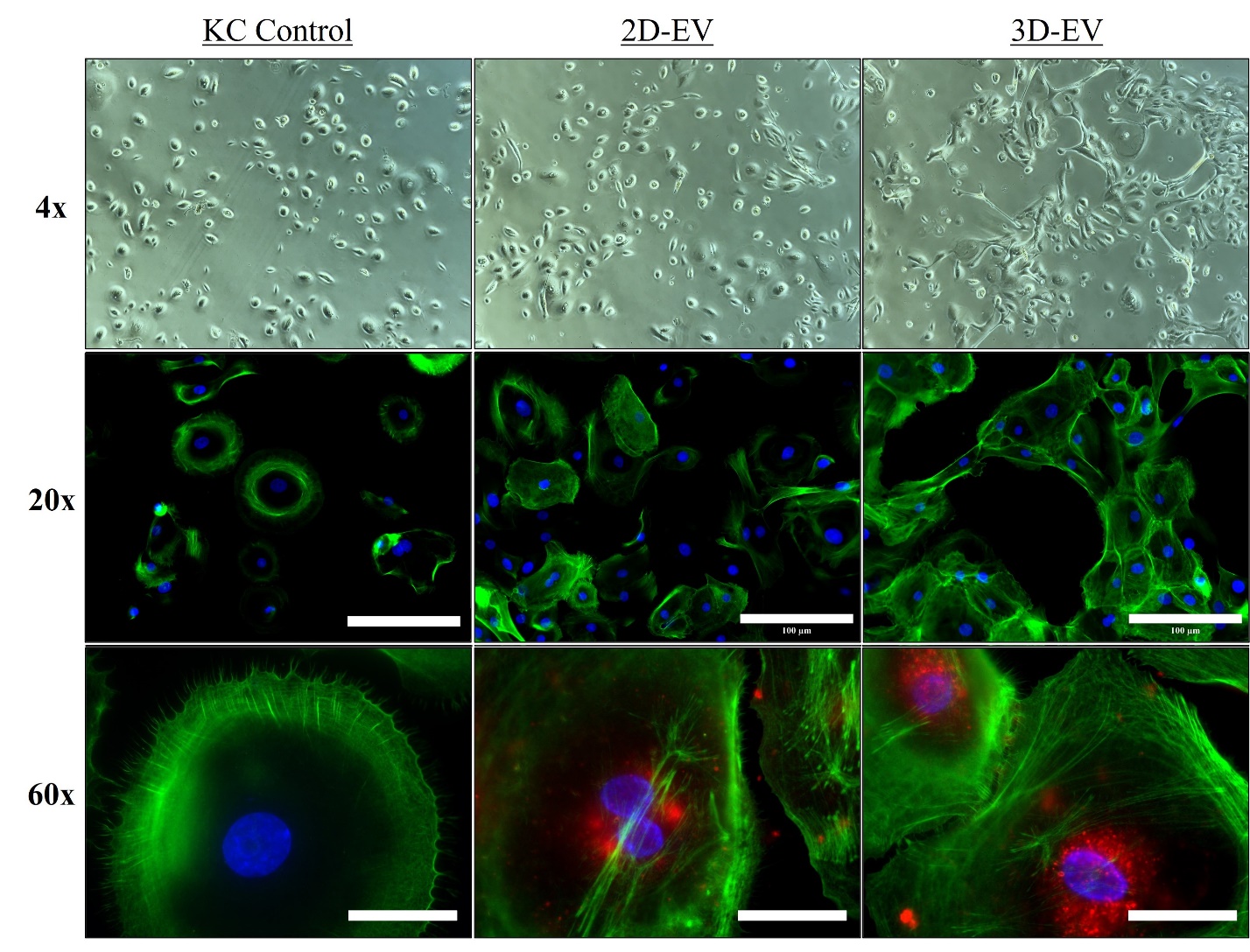
**

**Supplemental Figure 5: Comparative KC Morphological Changes with/without DiI-labeled EVs.** ASC-EVs were isolated from both 2D and 3D systems, labeled with a lipophilic dye, DiI (*red*), and dosed into KC-GM at 250µg/mL for treatment of KCs and assessment of morphological changes. KC morphology under transmitted light (*top row*), low magnification (20x) of fluorescent images of KCs with phalloidin (*green*) and Hoechst (*blue*) to depict formation of cell sheets in 3D-EV treated group, and high magnification (60x) of DiI-labeled EVs (*red*) to assess perinuclear localization of EVs. Control KC group (*left column*) compared to 2D-EV treated (*middle column*) and 3D-EV treated (*right column*). Scale bar for 20x images = 100µm, for 60x images = 25µm.

**
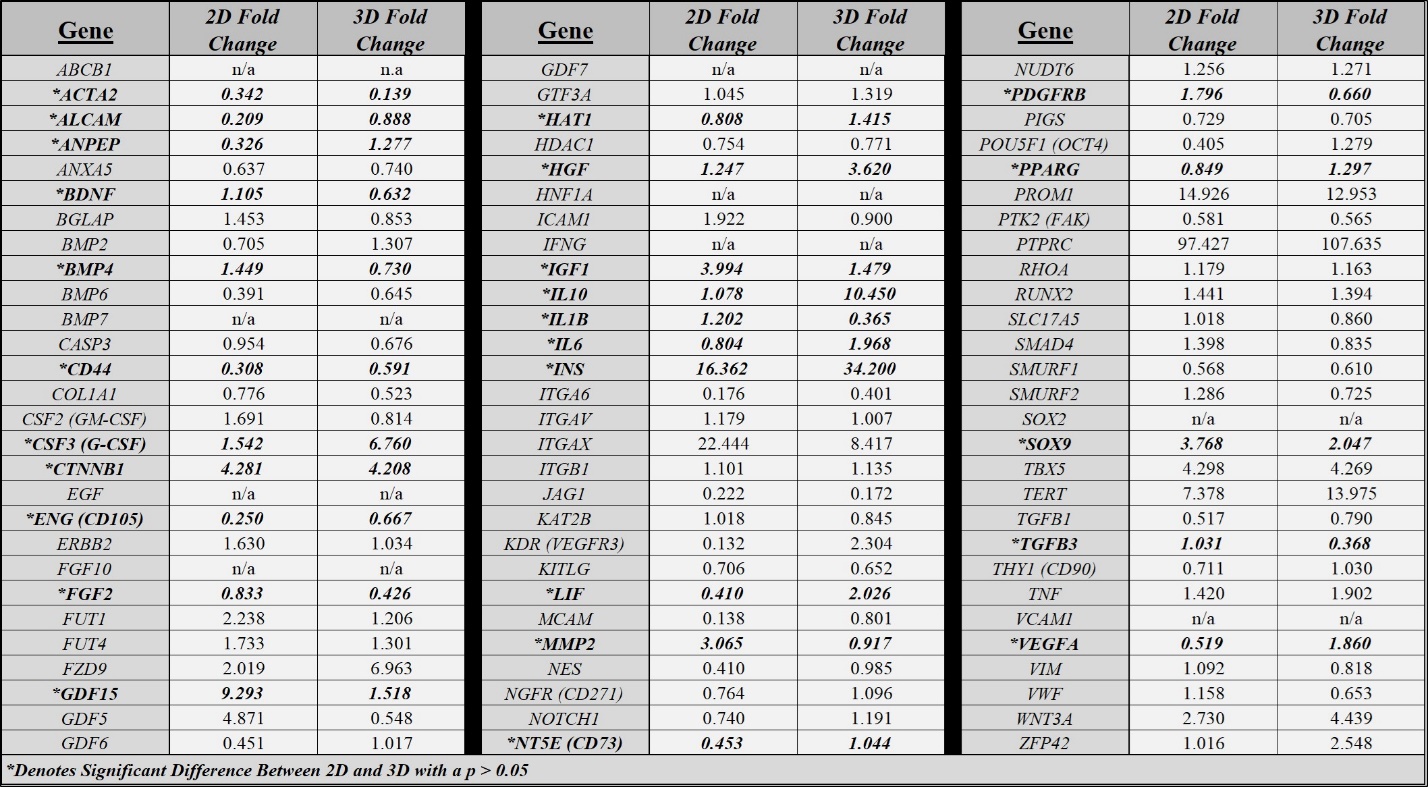
**

**Supplemental Table 1:** **Gene Expression of MSC Phenotype Profiling Array.** Genes marked “Bold” and with an “*” exhibited a significant difference between the relative 2D and 3D expression. Gene expression calculated via ΔΔCt methodology relative to baseline control KCs and endogenous control genes.


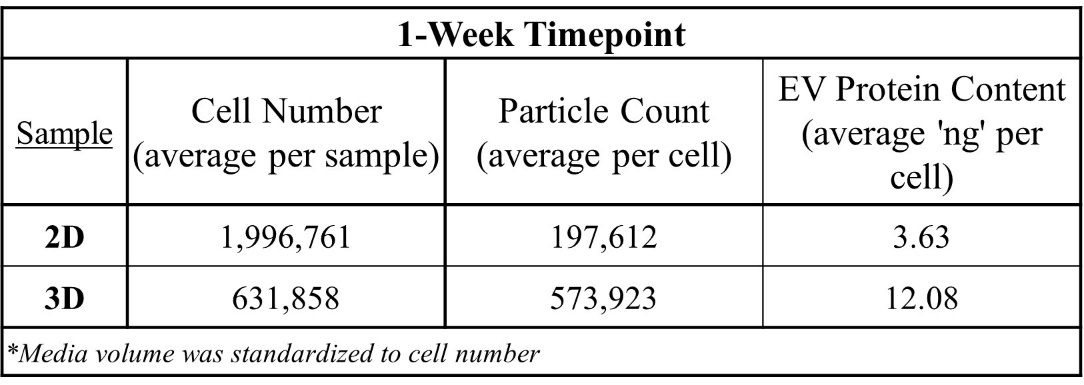


**Supplemental Table 2:** **Relative EV Particles and EV Protein per Cell.** Based on the cell numbers obtained with PicoGreen, the total EV particle counts obtained with NTA, and the total EV protein content obtained with QuickDrop, the relative production of EVs per cell was calculated. Only the 1-week timepoint was evaluated since this was the timepoint used for all the conditioned media experiments within this study.
